# An Overview of West African Traditional Soft Cheese: Processing, Safety, and Quality Characteristics

**DOI:** 10.1111/1541-4337.70301

**Published:** 2025-10-05

**Authors:** Mahounakpon Wilfried Tossou, Matthew Atongbiik Achaglinkame, Linda Dari, Carole Nadia Adjouavi Sossa‐Vihotogbé, Zsanett Bodor, Daniel Mörlein

**Affiliations:** ^1^ Department of Nutrition and Food Sciences University of Parakou Parakou Benin; ^2^ Department of Animal Sciences University of Göttingen Göttingen Germany; ^3^ University for Development Studies Tamale Ghana

**Keywords:** coagulation, milk, preservation, quality, safety, West African indigenous cheese

## Abstract

West African soft cheese (WASC) is widely consumed and nutritionally valuable across West Africa. It also sustains livelihoods, particularly among Fulani households, by providing regular income for women and strengthens household food security and autonomy. However, concerns persist about its safety and quality. A scoping review was conducted to synthesize evidence on WASC processing, safety, and quality. The findings showed that cow's milk (88.6%) and *Calotropis procera* (77.1%) were the predominant milk source and coagulant, respectively. Two research areas contribute to about one third of the total articles reviewed: alternative coagulants (22.9%) and partial substitution of cow's milk (11.4%). Among the various coagulants tested, only *Carica papaya* consistently achieved yields comparable to *Cal. procera*. Partial substitution with plant‐based milks differentially influenced yield and composition. Findings indicated frequent microbiological hazards in market‐sold WASC, with aerobic plate counts often exceeding limits and recurrent reports of coliforms and pathogens (e.g., *Escherichia coli*, *Salmonella*). In contrast, WASC produced under controlled laboratory conditions showed low aerobic counts and the absence of coliforms. In general, reporting quality was a major limitation, as most of the studies reviewed failed in reporting key processing parameters such as heating temperatures and times, coagulant concentration, and coagulation time, factors essential for assessing and reproducing cheese quality. In conclusion, this review underscores the urgent need for hygiene interventions across the value chain. Moreover, comprehensive research is needed to assess how different coagulants, extraction methods, heating conditions, and the alternatives could affect the yield, sensory characteristics, and overall quality of WASC.

## 1 Introduction

Milk and dairy products are important sources for ensuring good nutrition and livelihoods for millions of people around the world. Milk is mainly sold worldwide in the form of processed dairy products (OECD/FAO 2022). As milk is a highly nutritious and perishable product, it must be processed shortly after collection (FAO 2019). The dairy sector can contribute to the fight against malnutrition in vulnerable groups by optimizing production, processing, and marketing channels (Kunadu et al. 2019). Cheesemaking is one of the best processing methods to conserve the important nutrients of milk (Mazorra‐Manzano et al. 2018). There are diverse kinds of cheeses, classified by texture/moisture content (hard, semihard, and soft cheeses), maturation (unripened and ripened cheeses), coagulant type (rennet or acid), microorganisms used (bacteria or fungi), and the type or origin of milk (Almena‐Aliste and Mietton 2014; Gobbetti et al. 2018; Ouwehand 2021). These characteristics give cheeses specificity, which influences consumer preferences (Nyamakwere et al. 2021). Although the consumption and demand for cheeses with specific characteristics are not as developed in Africa as in Europe and other parts of the world, many kinds of cheeses are believed to be indigenous to Africa (McSweeney et al. 2004). Notable indigenous African cheeses include: *aoules* of Algeria; *fromage* of Madagascar; braided, *gibna*, and *mudaffara* of Sudan; *ayib* of Ethiopia; *mashanza* of the Democratic Republic of the Congo (formerly Zaire); and West African soft cheese (WASC; so called because of its identity with many West African countries, including the Republic of Benin, Burkina Faso, Ghana, Niger, Nigeria, and Togo (Figure 1), among others (O'Connor 1993).

**FIGURE 1:**
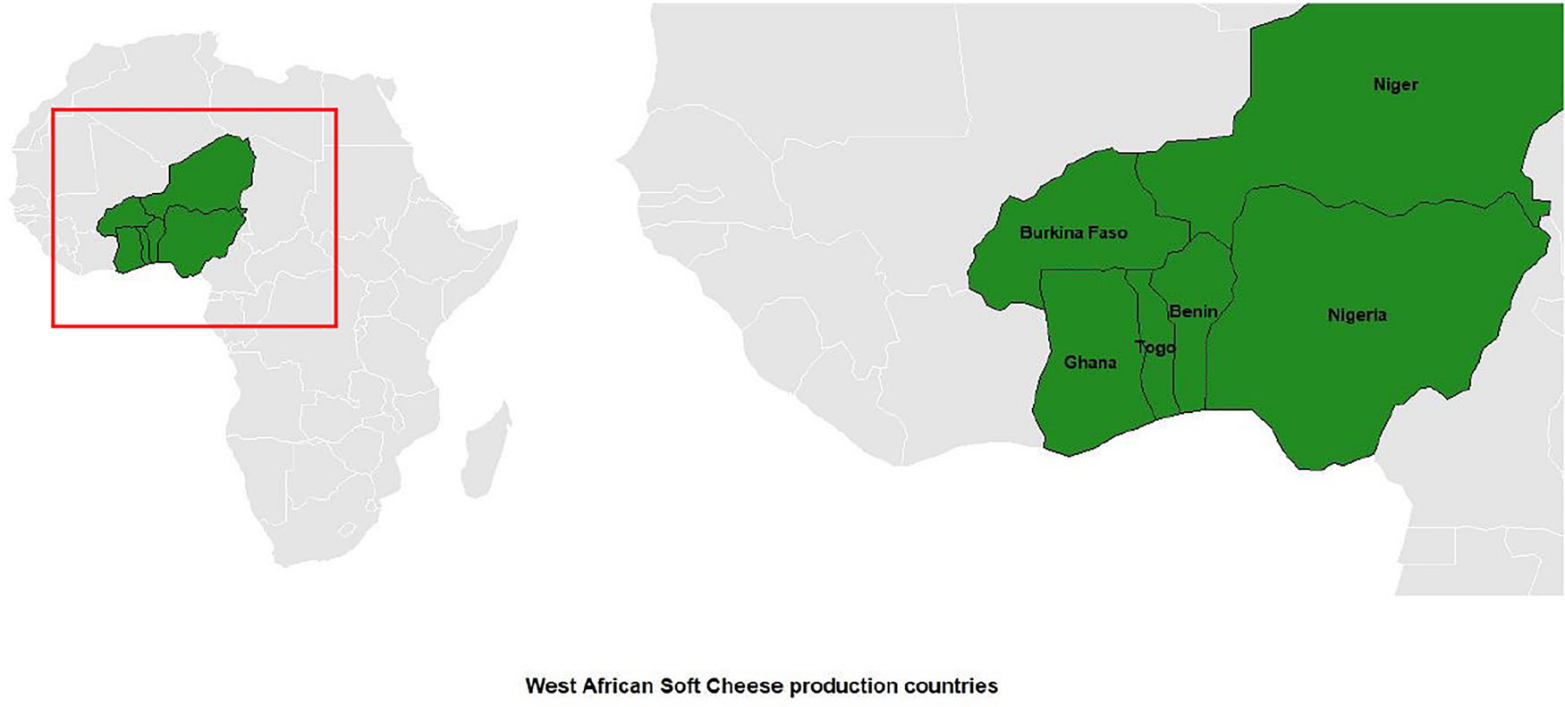
Countries with documented production of West African soft cheese in West Africa.

In Africa, cheese processing is mostly handled by small‐scale processors (largely women) at the home or farm level (O'Connor 1993). Such is particularly the case for WASC processing; its production is still largely a traditional craft and involves the use of rudimentary equipment (Adegoke et al. 1992). The traditional cheese, generally made from cow's milk, is mainly produced by Fulani women and sold along major streets and local markets. The Fulani people, also known as Fulbe, are one of the largest ethnic groups in the Sahel and West Africa, widely dispersed across the region. They are mainly pastoralists, making them the ethnic group with the largest nomadic pastoral community in the world (Mba 2022). The Fulani community is locally the main supplier of milk and local cheese across Western Africa (Agboola et al. 2020). Within Fulani households, milk is both a dietary staple and a primary economic resource. Men typically undertake the milking; the milk is then transferred to women, who determine the share reserved for household consumption and the portion processed into WASC for sale (Chabi Toko 2016). Sometimes, the women's role extends to the milking of the animals (Fayama et al. 2024). Women control processing, marketing, and the resulting income, which constitutes a substantial proportion of household earnings (Chabi Toko 2016).

Although WASC processing is common to most West African countries, it can vary from one processor to another and from one country to the other, depending on local know‐how and culinary traditions (FAO 2019). For example, Dossou et al. (2022) reported the usage of different parts of *Cal. procera* as coagulants by different processors in Benin: about 66% of processors used both the leaves and stems, while 32% and 2% made use of the stems and leaves as coagulants, respectively. Furthermore, coloring of cheese using sorghum extracts is practiced in Benin (Dossou et al. 2022; Komagbe et al. 2023), but such is not the case in Ghana.

The WASC is a nutritious food and a good source of protein, fat, vitamins, and minerals such as calcium and phosphorus. It is widely used as a substitute for meat or fish, or in combination with them in various food recipes, especially for low‐income people (Aboudoulaye and Kaya 2020; Adeyeye et al. 2021). This traditional cheese is classified as an unripened soft cheese with a high moisture content of about 50%–60%, which makes it highly perishable (McSweeney et al. 2004).

The most important step in cheese processing is the coagulation of milk, obtained by adding enzymes to it (Mazorra‐Manzano et al. 2018). Animal rennet, plant‐derived coagulants, coagulants of microbial origin, and genetically engineered chymosin are the four types of enzymes used in cheesemaking worldwide (Dossou et al. 2022). In West Africa, coagulants of plant origin are the most widely used in cheese production (Aboudoulaye and Kaya 2020). Sodom apple (*Cal. procera*) parts (sap, leaves, and/or stems), believed to be important for flavor development, are the most used milk‐coagulating agents for WASC processing. The formulation and development of new products with new and acceptable sensory characteristics are among the principal areas of innovation in cheese processing (García et al. 2012). Processors consider that cheese made with *Cal. procera* has a sweeter flavor than cheeses made with other coagulants such as *Car. papaya* and *Citrus* spp. (Aboudoulaye and Kaya 2020; Rayanatou et al. 2017). However, this claim of usage of *Cal. procera* as a coagulant resulting in WASC with more preferred sensory traits than other coagulants of plant origin has not yet been established. To ascertain the veracity, generality, or otherwise of this claim, a thorough review of literature is required. The review of literature is also important for coagulant effect comparison in terms of WASC yield, nutritional quality, and microbial safety. Across West Africa, cow's milk is the conventional milk for WASC processing (O'Connor 1993). However, alternative milk sources, largely of plant origin, are currently being explored for the partial replacement of cow's milk in WASC production. It is, however, yet to be established how this innovation of partial substitution impacts the yield, physicochemical quality, microbial safety, and sensory appeal of WASC. The quality of the milk, cooking time, temperature, coagulation conditions, and the coagulant used affect the quality of the cheese (Dossou et al. 2022). The impact of these processing conditions and milk quality may be more pronounced than perceived, especially from one processor to another and from one country to the other, especially when WASC processing is mainly still at the individual level. In Western Africa, WASC processors, who largely live in remote areas without electricity, lack cold storage facilities to ensure a longer shelf life of the cheese (Achaglinkame et al. 2023). Thus, when WASC is left at ambient temperature, it undergoes considerable chemical changes, and the shelf life does not exceed three days (Belewu et al. 2005). That is why some studies have tested the application of chemical preservatives or different types of coagulants (*Citrus* spp.) as an alternative preservative way of increasing the shelf life of the product in the absence of cold storage facilities. Although some of these preservatives (sorbic acid, sodium benzoate, ginger, and/or garlic extract) are effective in inhibiting bacteria such as mesophiles and coliforms, they are not readily available (physically and financially inaccessible) to local cheese processors in West Africa (Adetunji et al. 2008). Although some research efforts have been invested in the WASC, the quantum, depth, and scope of work done so far are unknown. This review thus aims to provide a comprehensive synthesis of existing research that explored and addressed various aspects related to the production, safety, and quality of WASC, in order to highlight the needed knowledge and reveal the key target areas for further research. Findings and recommendations of this review will ultimately help to ensure safe and quality WASC that not only meets consumers’ sensory preferences and nutritional needs but also increases processors’ and vendors’ incomes for improved livelihoods.

## 2 Materials and Methods

### 2.1 Type of Systematic Review

This study employed a scoping type of review to identify the evolution of research about the WASC, the location of the research, and research gaps for future studies. Research about WASC is evolving, and this review identified the state of research so far, the type of study, key findings, topical and methodological gaps/grey areas, and possible recommendations.

### 2.2 Literature Search

A systematic literature search of the studies made on WASC was performed to summarize the state of the art. Searches were conducted between January 7, 2023 and October 1, 2024 in Scopus, Web of Science, and PubMed, with supplementary searches in Google Scholar and ResearchGate using common and traditional names associated with WASC: “West and African and soft and cheese” or “Peulh and cheese” or “West and African and cottage and cheese” or “West and African and traditional and cheese” or “Wagashi and Gassire” or “Wagashie” or “Wangashi” or “Warankashi” or “Warankasi” or “Warankashi” or “Wara” or “Waghazi” or “Woagachi.” Searching was also done within the article title, abstract, and keywords (Appendix 1).

### 2.3 Inclusion and Exclusion Criteria

Studies were purposively selected and included if they were based on the production and evaluation of the WASC quality, by partial substitution of cow's milk either with other animal milks or with plant‐based milk alternatives, and were published in English. Literature that covered WASC produced using Sodom apple (*Cal. procera*) extracts and/or other coagulants, storage and preservation, and sensory properties was also included. In contrast, studies were excluded if they evaluated the complete substitution of cow's milk. There were no restrictions on the year of publication. Published materials such as journal articles, books, book chapters, conference proceedings, and theses, once they met the selection criteria, were included. Only published works that conducted actual experiments or surveys were included. The articles considered and finally included were those available in full text.

The papers’ selection process consisted of four steps. First, the search results were compiled and deduplicated, followed by the title screening. Then, the abstract screening was performed, followed by the full‐text screening. Two independent reviewers screened each search result. Findings were reported according to the Preferred Reporting Items for Systematic reviews and Meta‐Analyses (PRISMA) (Page et al. 2021). Descriptive statistics were performed using R software (R Core Team 2024) and Jamovi (The Jamovi Project 2024).

## 3 Results and Discussion

### 3.1 Study Characteristics

The search yielded 525 papers, of which 385 were screened after duplicate removal. Screening and application of inclusion criteria produced 70 studies (of which 67 were indexed) used in this review. Figure 2 shows the results of the literature selection process.

**FIGURE 2:**
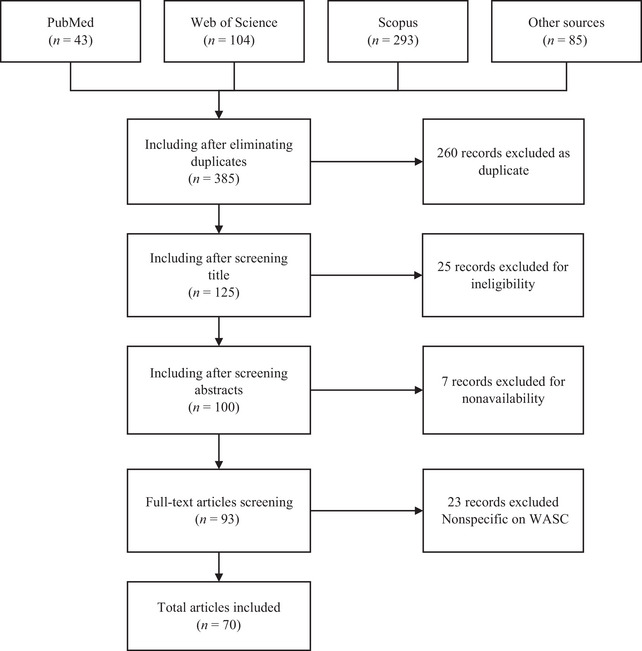
Flowchart of the literature selection process.

The 70 included papers were published in the period 1983–2024, with most of them having been published between 2017 and 2022 (*n* = 28, 40%) and 2011–2016 (*n* = 21, 30%), revealing an increased research interest within that period. Most of the studies were experimental (*n* = 45, 64.3%) or observational (i.e., sampling and/or survey) (*n* = 22, 31.4%), showing more interest in self‐producing the cheese for research than sampling from elsewhere. Studies were mainly published by authors from Nigeria (*n* = 47, 67.1%), Ghana (*n* = 10, 14.3%), and Benin (*n* = 6, 8.6%), using the first authors' countries of affiliation. Authors were also from Algeria (*n* = 2, 2.9%), Turkey (*n* = 2, 2.9%), Burkina Faso (*n* = 1, 1.4%), Kenya (*n* = 1, 1.4%), and Ethiopia (*n* = 1, 1.4%). This clearly showed that Nigeria quantitatively did more research into cheese than all the other countries combined. We found two areas of research contributing to about one third of the total articles reviewed: use of alternative coagulants to *Cal. procera* (*n* = 16, 22.9%) and partial substitution of cow's milk (*n* = 8, 11.4%). The alternative coagulants included *Citrus* spp. (*n* = 11), *Car. papaya* (*n* = 4), *Moringa oleifera* (*n* = 2), steep water from cereals (maize, millet, sorghum) (*n* = 2), *Plumeria alba* (*n* = 1), *Pergularia tomentosa* (*n* = 1), *Tamarindus indica* (*n* = 1), *Mangifera indica* (*n* = 1), and CaCl_2_ (*n* = 1). Plant‐based drinks or milk alternatives, considered as plant‐based milk in this review, mainly from coconut (*Cocos nucifera*) (*n* = 3), soya beans (*Glycine max*) (*n* = 3), Tiger nut (*Cyperus esculentus*) (*n* = 1), Bambara groundnuts (*Vigna subterranea*) (*n* = 1), and cocoa powder solution (*n* = 1), were used as a substituent for cow's milk. Table 1 shows the number and percentage of articles related to the main research.

**TABLE 1:** Overall studies’ features (publication date, type of study, affiliation country of the first author, main aim of the research) of the articles considered in this review.

Study characteristics	*N* (%) (total *n* = 70)
Year of publication	
1983–1999	6 (8.6%)
2000–2010	9 (12.9%)
2011–2016	21 (30%)
2017–2022	28 (40%)
After 2022	6 (8.6%)
Type of study	
Experimental	46 (65.7%)
Observational (sampling and survey)	22 (31.4%)
Both	2 (2.9%)
Author affiliation	
Nigeria	47 (67.1%)
Ghana	10 (14.3%)
Benin	6 (8.6%)
Algeria	2 (2.9%)
Turkey	2 (2.9%)
Burkina Faso	1 (1.4%)
Ethiopia	1 (1.4%)
Kenya	1 (1.4%)
Research interests	
Alternative coagulants	16 (22.9%)
Partial substitution of cow's milk	8 (11.4%)
Safety, conservation	46 (65.7%)

### 3.2 General WASC‐Making Process

**FIGURE 3:**
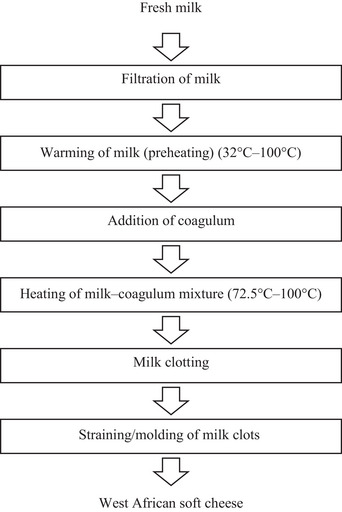
General West African soft cheese processing.

Generally, based on the articles reviewed, the preparation of WASC involves several steps that remain almost the same regardless of the country. First, the sourced cow's milk is filtered to get rid of the animal's fur and other impurities and warmed (Dossou et al. 2022). Plant coagulant extracts are then added to the warmed milk, and the temperature is maintained or elevated afterward till coagulation sets in. The coagulated milk is further cooked for a few minutes to facilitate whey expulsion and inactivate the coagulating enzyme (Adetunji and Salawu 2008; Akinloye and Adewumi 2014). The curdled milk is then scooped into specially designed cheese baskets to strain and mold into desired shapes. Figure 3 presents a general flow diagram illustrating the WASC preparation process based on the literature reviewed.

#### 3.2.1 Milk Sources

For decades, traditional dairy systems have dominated milk production in Africa and continue to supply substantial quantities to this day. These traditional systems contribute to more than 90% of the dairy ruminant population in sub‐Saharan Africa (Mattiello et al. 2018). The primary suppliers of milk are the Fulani herdsmen, who operate within rural communities (Chikpah et al. 2016; Mba 2022). In West Africa, cattle rearing is mostly extensive, especially during the dry season, when herds migrate in search of pasture. This migration is dictated by the availability of biomass, which is highly dependent on seasonal precipitation. Consequently, livestock production, particularly from grazing‐dependent ruminants, exhibits marked seasonality (FAO 2020). During the prolonged dry season, milk collection significantly diminishes, contributing to severe shortages (Achaglinkame et al. 2023). Given these seasonal challenges and the resulting scarcity of cow's milk, researchers have increasingly focused on exploring alternative sources of affordable, nutritionally rich milk to supplement the limited supply of cow's milk (Chikpah et al. 2016).

Among those alternatives, plant‐based milk derived from locally available, protein‐rich foods presents a viable solution (Okorie and Adedokun 2013). However, the production of plant‐based milk using legumes and oilseeds is not a new practice. Indeed, for both traditional and economic reasons, the use of dairy products has often been constrained, fostering interest in plant‐based substitutes that could serve as vehicles for probiotics or replace dairy milk in various products such as those derived from cereals, fruits, and vegetables (Granato et al. 2010). Plant‐based milk and its derivative products offer significant nutritional benefits for people of all ages due to their richness in proteins, essential minerals, and fatty acids, which are highly valuable for human health (Brough et al. 1993; Xie et al. 2023). Soya milk, among plant‐based alternatives, is the most comparable to cow's milk in protein amount and fermentation‐relevant functionality (Bolla 2015; Prado et al. 2008). Yet, micronutrient deficiencies (mainly calcium, vitamins B12 and B2, and iodine) need to be considered when fully replacing cow's milk with plant‐based alternatives (Kersting et al. 2025). Soya milk, like animal milks, can be coagulated to produce cheese and fermented to create yoghurt, offering high‐quality proteins, unsaturated fatty acids, dietary fibers, and isoflavones, along with antioxidant and phytonutrient properties that benefit human health (Ahmed et al. 2015). Recent studies have continued to highlight the growing scientific interest in soya milk as an alternative to cow's milk (Hussein et al. 2016; Jayarathna et al. 2021).

Soya milk has successfully been employed as a partial substitute for cow's milk in the production of WASC (Aworh et al. 1987; Chikpah et al. 2016; Hussein et al. 2016). Aworh et al. (1987) explored substitution levels from as low as 5% to as high as 20%. In contrast, Chikpah et al. (2016) and Hussein et al. (2016) investigated higher substitution ratios, with soya milk comprising between 25% and 75% of the total milk content. This integration could be a good way to alleviate the challenges posed by milk scarcity, particularly during the dry season, while providing a nutritious and sustainable alternative for local cheese production. Similarly, Okorie and Adedokun (2013) demonstrated the feasibility of partially substituting cow's milk with Bambara groundnut milk at levels ranging from 5% to 50%. Bambara groundnut has a chemical composition comparable to that of soybean, and its nutrient profile supports the growth of probiotics (Brough et al. 1993; Murevanhema and Jideani 2013).

Further diversification in WASC production has been explored with other plant‐based milk alternatives. Oni et al. (2020) studied the use of Tiger nut and coconut milk, achieving successful substitution levels of up to 50%. Balogun et al. (2016) and Okon and Ojimelukwe (2017) have also tested the incorporation of coconut milk in the production of WASC in ranges of 5%–30%. Even cocoa powder solution has also been incorporated into WASC production at levels up to 40% (Omobolanle et al. 2015). In terms of animal milk substitutes, sheep's and goat's milk have not been commonly used for partial replacement in WASC production; instead, they have primarily been used as complete substitutes, utilized either individually or separately as viable alternatives to cow's milk (Akinloye and Adewumi 2014; Ogunlade et al. 2019; Ogunlade et al. 2020).

#### 3.2.2 Milk Heating Conditions

**FIGURE 4:**
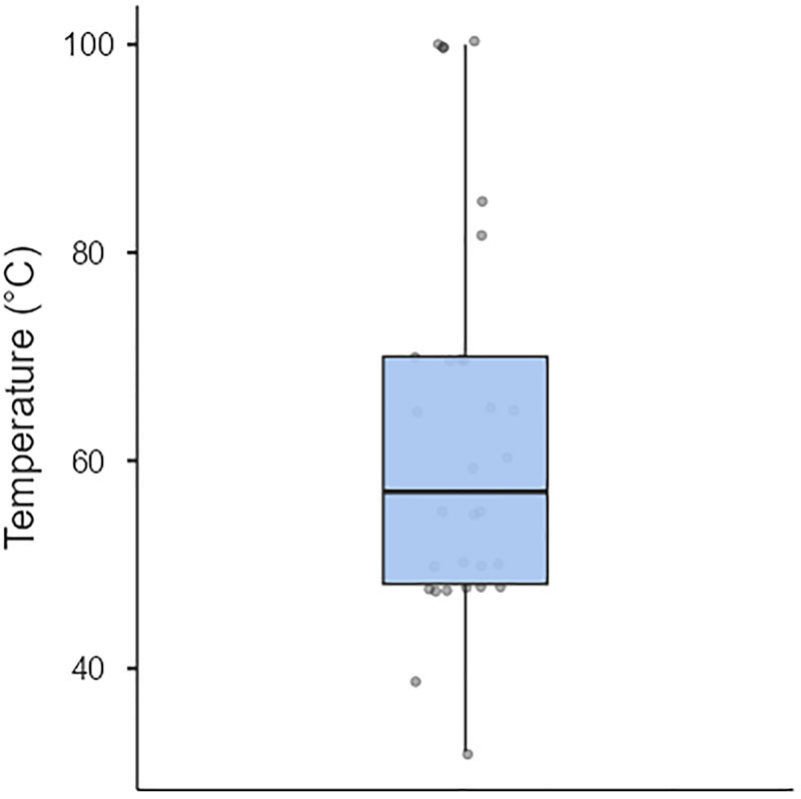
Variability in milk preheating temperature (*n* = 29).

The process of preparing WASC involves multiple stages of heating, each playing a crucial role in ensuring proper coagulation and enhancing the overall quality of the cheese. The initial step typically includes preheating/warming the milk to a specific temperature before introducing the coagulant. This preheating phase is essential as it facilitates the coagulation process by optimizing the milk's condition, allowing the coagulant to act. The temperatures utilized during this stage can vary remarkably, as reported in various studies. These reported temperatures range from a minimum of 32°C to as high as 100°C, with the median temperature around 57°C (Figure 4) (Adetunji et al. 2008; Akinloye and Adewumi 2014; Ayeni et al. 2014; Abebe and Emire 2020; Aboudoulaye and Kaya 2020; Ayodeji et al. 2020; Adesina et al. 2022; Balogun et al. 2016; Benyahia et al. 2016; Badmos et al. 2018; Bankolé et al. 2019; Benyahia et al. 2021; Chikpah et al. 2014; Chikpah et al. 2016; Dari and Appah 2023; Hussein et al. 2016; Joseph and Akinyosoye 1997; Koranteng et al. 2021; Mahami et al. 2012; Ogundiwin and Oke 1983; Okorie and Adedokun 2013; Omobolanle et al. 2015; Olorunnisomo and Adewumi 2016; Orhevba and Taiwo 2016; Okon and Ojimelukwe 2017; Ogunlade et al. 2020; Oni et al. 2020; Tossou et al. 2018). The variation in preheating temperatures could be attributed to diverse factors such as differences in traditional practices, milk composition, and the type of coagulant used. While almost half (49%) of the experimental studies reviewed failed to indicate the duration of preheating, 240–300 min (incubation) (Abebe and Emire 2020) and 30–40 min (Orhevba and Taiwo 2016) were, respectively, reported for the two extreme temperatures, 32°C and 100°C. Reporting both the preheating temperature and preheating time not only enhances reproducibility but could also have quality and economic implications. The use of temperatures above 60°C for preheating of the milk plays an important role in milk pasteurization or sterilization to improve its safety (Johnson et al. 1990). The second phase of heating involves cooking the coagulated milk at temperatures typically ranging between 72.5°C and 100°C for a while (10–65 min) (Adegoke et al. 1992; Adetunji et al. 2008; Akinloye and Adewumi 2014; Aboudoulaye and Kaya 2020; Balogun et al. 2016; Benyahia et al. 2016; Ogundiwin and Oke 1983; Ojedapo et al. 2014; Okon and Ojimelukwe 2017; Oni et al. 2020). This step is crucial for facilitating the expulsion of whey. Additionally, it serves the purpose of inactivating the coagulating enzyme. Despite its importance, specific details regarding these temperature and time ranges are seldom highlighted in the literature. For example, only 22% and 9% of the experimental studies reported cooking temperature and cooking time, respectively. Nonetheless, understanding and controlling this heating phase is essential for achieving consistent results in the quality of WASC.

#### 3.2.3 Coagulants and Their Extraction

##### 3.2.3.1 Coagulants

Milk coagulation can occur through enzymatic action, acidification, or a combination of both processes. Animal‐sourced rennet has, by far, been the most conventional coagulating agent for cheesemaking. However, growing animal welfare and sustainability awareness, coupled with ethical, religious, and consumer demands, have occasioned the exploration of microbial and plant‐based alternatives in cheesemaking (Buele et al. 2024). The use of plant‐based coagulants for milk coagulation is especially prevalent in the production of artisanal cheeses in western Africa, which are traditionally made by hand (Mohsin et al. 2024). This method is integral to the preparation of WASC, where the incorporation of plant‐based coagulants plays a vital role.

One key group of enzymes from the plant‐based coagulants are cysteine proteases, also known as thiol‐proteases (Mazorra‐Manzano et al. 2013; Shah and Mir 2019). In addition to their catalytic specificity, cysteine proteases exhibit a strong tolerance to varying pH levels and temperatures, making them highly suitable for diverse dairy applications (Ramos et al. 2013).

Among the diverse plant‐based coagulants available for utilization in milk coagulation, the Sodom apple plant (*Cal. procera*) (Plate 1) is the most used for WASC. *Calotropis procera* contains an enzyme known as “calotropin,” which acts as a cysteine protease coagulant. This enzyme is instrumental in promoting milk‐clotting activity, a key determinant of the suitability of plant proteases for cheese production (Benyahia et al. 2016; Shah and Mir 2019). Plant‐derived proteases used for milk coagulation can be sourced from various parts of plants, including fruits, leaves, flowers, seeds, latex, and rhizomes (Shah et al. 2014). In the case of *Cal. procera*, different parts such as the leaves, stems, and latex (milky sap) have been employed as coagulants (Table 2). Beyond its role in cheesemaking, *Cal. procera* is a multipurpose plant that provides numerous provisioning ecosystem services and has long been utilized in traditional medicinal systems in regions including North Africa, the Middle East, and South and Southeast Asia (Al Sulaibi et al. 2020). Research has documented the presence of a wide array of bioactive compounds in the plant, such as flavonoids, tannins, terpenoids, saponins, alkaloids, steroids, and cardiac glycosides (Al‐Rowaily et al. 2020; Mossa et al. 1991; Moustafa et al. 2010). The leaf extracts of *Cal. procera* have been found to contain significant amounts of fatty acid ethyl esters (21.4%), palmitic acid esters (10.2%), linoleic acids (7.4%), and amino acids (8.1%) (Pattnaik et al. 2017). However, concerns have been raised regarding the potential toxicity of *Cal. procera*, as it has been shown to cause acute toxicity in plant and animal cells, including humans (Kaur et al. 2021; Waikar and Srivastava 2015). The relationship between its bioactivity and toxicity remains underexplored, although some studies have suggested it may induce cardiotoxicity and hepatotoxicity (De Lima et al. 2011). Conversely, a safety assessment conducted by Mossa et al. (1991) revealed that the use of aerial parts of the *Cal. procera* plant (ethanol extract) in single doses as high as 3 g/kg did not exhibit toxic effects in guinea pigs until the treatment of >90 days was provided. Additionally, Bezerra et al. (2017) reported that latex proteins from the plant, when orally administered to mice, did not produce adverse immunological reactions even at doses of up to 5000 mg/kg. However, intraperitoneal administration at a dose of 150 mg/kg resulted in mortality within 1 h. Moreover, Abebe and Emire (2020) demonstrated that administering leaf extracts of *Cal. procera* at a dose of up to 134.2 mg/kg did not induce significant behavioral changes (e.g., alertness, aggressiveness, irritability) or affect gross physical appearance (e.g., fur condition, general cleanliness) in albino mice in comparison to control groups. Importantly, a study by Tossou et al. (2018) showed that the various phytochemicals, such as alkaloids, flavonoids, tannins, cardiac glycosides, phenols, saponins, and steroids, present in *Cal. procera* were not detected in the resulting cheese or whey. They concluded that the WASC and/or whey processing denatures the phytochemicals, thereby ensuring the safety of the products (Tossou et al. 2018).

**TABLE 2:** Coagulants, parts, and quantity used in West African soft cheese preparation.

Type of coagulant	Parts	Mode of utilization	Quantity of coagulant (g per 1 L of milk)	References
*Calotropis procera*	Dried leaves	Crude enzyme	1; 3; 5	Abebe and Emire 2020
	Fresh leaves	Crude enzyme	40	Aboudoulaye and Kaya 2020
	Fresh leaves	Purified enzyme	6	Aboudoulaye and Kaya 2020
	Fresh leaves	Extract	2.5	Aworh and Egounlety 1985; Aworh et al. 1987; Adesina et al. 2022; Joseph and Akinyosoye 1997
	Leaves and stems	—	60	Adetunji and Chen 2011
	Fresh leaves	—	3.2	Adetunji and Salawu 2008
	Fresh leaves	—	17.5	Akogou et al. 2018
	Fresh leaves	—	35	Anihouvi and Kesenkaş 2022
	Fresh leaves	Extract	50	Ayeni et al. 2014
	Stem	—	10	Badmos et al. 2017
	Fresh leaves	—	10	Badmos et al. 2018
	Fresh leaves	—	15	Balogun et al. 2016; Oni et al. 2020
	Fresh leaves	—	20	Benyahia et al. 2016
	Dried leaves and stem	—	2; 5; 7	Chikpah et al. 2014
	Fresh leaves and stem	—	18.7; 46.7; 65.4	Chikpah et al. 2014
	Fresh leaves	Extract	16.7	Dari and Appah 2023
	Fresh leaves and stem	Extract	90.9	Okon and Ojimelukwe 2017
	Latex	—	2.5	Koranteng et al. 2021; Orhevba and Taiwo 2016
	Fresh leaves	Extract	5; 10; 15; 20; 25	Ogundiwin and Oke 1983
	Fresh leaves	Extract	4	Ojedapo et al. 2014; Ogunlade et al. 2019
	Fresh leaves	Extract	111.1	Okorie and Adedokun 2013
	Latex	—	5	Orhevba and Taiwo 2016
*Citrus* spp.	Fruits	Juice	4	Ogunlade et al. 2019
	Fruits	Juice	5	Ayodeji et al. 2020
	Fruits	Juice	12.4	Adetunji 2008; Adetundji 2011
	Fruits	Juice	16.5	Adetunji and Chen 2011
	Fruits	Juice	22.5	Adetunji et al. 2008
	Fruits	Juice	25	Olorunnisomo and Adewumi 2016
	Fruits	Juice	50	Dari and Appah 2023; Orhevba and Taiwo 2016
	Fruits	Juice	100	Orhevba and Taiwo 2016
	Fruits	Juice	111.1	Hussein et al. 2016
*Carica papaya*	Fresh leaves	—	3.2	Adetunji and Salawu 2008
	Fresh leaves and stems	Extract	4	Ogunlade et al. 2020
*Moringa oleifera*	Seeds	Concentration	1.5	Adesina et al. 2022
	Seeds	Extract	75; 100	Orhevba and Taiwo 2016
*Tamarindus indica*	Fruits	Juice	33.33	Ojochogu et al. 2022
Calcium chloride	—	—	50	Ayeni et al. 2014
*Mangifera indica*	Unripe fruits	Juice	25	Olorunnisomo and Adewumi 2016
*Pergularia tomentosa*	Dried leaves	Extract	136	Benyahia et al. 2021
Cereals (sorghum, maize, millet)	Steeped water	—	4	Ogunlade et al. 2020
Maize	Steeped water	—	33.33	Ojochogu et al. 2022
Vegetarian rennet		Solution (1 rennet tablet:50 mL deionized water)	16.7	Dari and Appah 2023

*Calotropis procera*, despite its significant applications in traditional medicine and the cheesemaking industry, has not yet been cultivated on a commercial scale (Adetunji and Salawu 2008; Akinloye and Adewumi 2014). This limited cultivation raises concerns about its future availability. If the plant were to face extinction or a marked decline in its population due to adverse climatic conditions, increased harvesting pressure, or the discovery of new, valuable applications, the cheese processors would be forced to seek alternative coagulant sources. The situation would be further exacerbated if there were a surge in demand driven by its varied benefits (Akinloye and Adewumi 2014). Consequently, the need for sustainable and effective alternatives has become a research interest in West Africa, focusing on identifying viable substitutes that can match or surpass the efficacy of *Cal. procera*. This review highlights that 22.9% of the selected studies investigated potential alternative coagulants (Table 1). These efforts encompass investigations into various plants that could serve as suitable replacements (Adesina et al. 2022; Adetunji 2008; Adetunji et al. 2008; Adetunji and Chen 2011; Adetunji and Salawu 2008; Akinloye and Adewumi 2014; Ayeni et al. 2014; Ayodeji et al. 2020; Bankolé et al. 2019; Benyahia et al. 2021; Dari and Appah 2023; Hussein et al. 2016; Ogunlade et al. 2019; Ojochogu et al. 2022; Olorunnisomo and Adewumi 2016; Orhevba and Taiwo 2016).

Citrus (*Citrus* spp.)—such as lemon, lime, and papaya (*Car. papaya*)—are the most utilized plant‐based coagulants as substitutes for *Cal. procera* in the preparation of WASC. The fruit juices of *Citrus* spp. are particularly valued for their high citric acid content, responsible for milk coagulation by acidification. Citric acid functions by lowering the milk's pH (∼6.6–6.8) toward the isoelectric point of casein (∼4.6), which destabilizes casein micelles, the primary protein complexes responsible for the structural integrity of milk (Goff et al. n.d.; Fachraniah et al. 2019). This pH reduction prompts the coagulation of casein and also facilitates the denaturation of whey proteins, further aiding the formation of a solid curd (Fachraniah et al. 2019). An additional advantage of using *Citrus* spp. as coagulants lies in their inherent antimicrobial properties. The acidic environment created by citric acid is not only effective for curd formation but also effective as a natural inhibitor of various pathogenic microorganisms such as Enterobacteriaceae (Adetunji et al. 2008). This property could enhance preservation and safety in cheese production, supporting the quality and shelf life of the final product (Adetunji et al. 2008; Fachraniah et al. 2019). The utilization of lemon juice in cheesemaking has been documented, underscoring its efficacy in curd formation (Bhagiel et al. 2018; Wibowo et al. 2020).

*Carica papaya* leaves (Plate 2) have been traditionally employed for their proteolytic properties, specifically due to the enzyme papain. Papain, an active member of the cysteine protease family, possesses a globular structure composed of a single polypeptide chain with three disulfide bridges and a crucial sulfhydryl group required for its enzymatic activity. This enzyme has proven versatile, extending its applications beyond cheesemaking to other areas such as medicine and the broader food processing industry (Babalola et al. 2023). Within the context of cheesemaking, papain is effective in facilitating the coagulation of milk proteins, contributing to the formation of curd across various types of cheese production (Aini et al. 2019; Fachraniah et al. 2019; Li et al. 2022). However, employing these alternatives in cheesemaking could present certain challenges. The excessive proteolytic action of papain or the high acidity from citrus fruits, when not carefully controlled, could result in cheese with undesirable sensory characteristics such as bitterness or an overly tangy flavor profile (Mohsin et al. 2024). Excessive proteolysis over‐hydrolyzes caseins, generating hydrophobic peptides that, when not adequately degraded, accumulate and give a bitter or undesirable taste (Fallico et al. 2005; Liburdi et al. 2019; Nicosia et al. 2022). Therefore, addressing these issues is essential for their successful application in WASC processing, ensuring that their use yields favorable sensory attributes while maintaining the quality and safety of the product.

Moringa (*Mo. oleifera*) seed extract, a good source of protein, calcium, and bioactive compounds, has emerged as another promising alternative coagulant, containing milk‐coagulating enzymes useful for cheesemaking (El‐Siddig et al. 2018; Fguiri et al. 2024; Putri et al. 2022). Wang et al. (2021), as cited by Yang et al. (2025), identified these enzymes as aspartic‐type endopeptidases, which function optimally at 60–65°C and a pH of 5 (Terefe et al. 2017; Wang et al. 2021). These enzymes have also been reported to exhibit good pH and thermal stability, suitable characteristics for cheese processing (Wang et al. 2021). Moringa seed extract has been used as a coagulant for buffalo's milk cheese (Yang et al. 2025), camel's milk cheese (Fguiri et al. 2024), and WASC (Adesina et al. 2022; Orhevba and Taiwo 2016). Tamarind (*T. indica*) fruits (Ojochogu et al. 2022) and unripe mango (*Ma. indica*) fruits (Olorunnisomo and Adewumi 2016) have also been explored for their milk coagulation properties. The acidic pulp of tamarind can effectively lower the pH of milk, leading to the denaturation and coagulation of casein proteins (Ojochogu et al. 2022). Tamarind has also been reported to contain polysaccharides that undergo interparticle bridging to facilitate coagulation and flocculation (Sofiavizhimalar et al. 2022). Tamarind has, therefore, been used as a coagulant in the processing of WASC (Ojochogu et al. 2022) and paneer (Indian cheese) (Shinde et al. 2025), in the treatment of dairy industry wastewater (Sivakumar et al. 2014; Sofiavizhimalar et al. 2022), as well as in the clarification of turbid water (Zainol and Fadli 2020; Zainol et al. 2021). Additionally, other sources such as steeped water from some cereals such as maize, sorghum, or millet (Ogunlade et al. 2019; Ojochogu et al. 2022) and calcium chloride (Ayeni et al. 2014) have also been used to coagulate milk during the processing of the cheese. Steeped water from plant materials has been reported to contain active extracted compounds that are able to disrupt and coagulate suspended particles (Oboh 2007). The exploration of alternative milk and coagulants underscores a diverse range of options available to cheese‐makers, providing essential flexibility and adaptability in the search for sustainable and effective WASC production practices. These alternatives may help mitigate issues related to the limited availability of resources in the context of climate change. Although the initial studies on these alternatives have shown promise, there remains significant room for further and more comprehensive research.

##### 3.2.3.2 Extraction methods

Plant‐based coagulants exhibit significant variability, not only in their chemical composition and enzymatic activity but also in how their active components interact with milk during the coagulation process. This variability makes it essential to consider that the conditions under which these coagulants are extracted and prepared can differ from one plant source to another (Mohsin et al. 2024). In most studies involving the Sodom apple (*Cal. procera*), cow's milk was employed as a solvent to extract the coagulant enzyme. However, a few investigations have utilized water as an extraction agent (Adetunji and Salawu 2008; Aboudoulaye and Kaya 2020; Oladipo and Jadesimi 2013). In contrast, other studies opted for a simpler method where plant parts were directly pounded and added to the milk without using a solvent (Opeyemi 2019; Balogun et al. 2016).

Aboudoulaye and Kaya (2020), in their work, which aimed to characterize and standardize the cheese's processing procedures, used crude as well as purified extracts of Sodom apple to coagulate the milk. Typically, leaves (*Cal. procera* and *Car. papaya*) and stems (*Cal. procera*) were crushed or pounded and mixed with the chosen solvent to maximize enzyme release. When using other plant‐based coagulants, such as *Mo. oleifera* dried seeds as coagulants, Adesina et al. (2022) and Orhevba and Taiwo (2016) ground the seeds and mixed them with water. Tamarind (*T. indica*) was soaked for 1 h and sieved for use as a coagulant (Ojochogu et al. 2022). Dossou et al. (2022) found six distinct methods of processing WASC in Benin by interviewing 165 experienced and active cheese processors from the municipalities of Dassa and Nikki. These methods varied based on the *Cal. procera* part (sap, stem, leaf) and extraction solvent (water, milk) used: (i) adding the *Cal. procera* sap to the milk being heated to coagulate it; (ii) using the filtrate of the mixture of a small quantity of milk and crushed leaves and stems to coagulate the mainstream milk; (iii) using the filtrate of the mixture of a small quantity of water and crushed leaves and stems as a coagulant for the mainstream milk; (iv) using the filtrate of the mixture of a small quantity of milk and crushed stems as a coagulant; (v) using the filtrate of the mixture of a small quantity of water and crushed stems as a coagulant; and (vi) adding directly crushed leaves and stems to the mainstream milk as a coagulant and then skimming off the leaf and stem rubbles.

In that study (Dossou et al. 2022), method 2 using the filtrate of crushed leaves and stems mixed with milk was the most commonly employed technique among cheese processors. These findings emphasize that a range of techniques exist for extracting and applying plant‐based coagulants. More comprehensive research is needed to assess how different coagulants used, as well as the extraction methods, could affect the quality and sensory characteristics of WASC. Table 2 provides a summary of the types of coagulants used in literature, along with the specific plant parts and quantities employed in the WASC‐making process. The variability in how the quantity of coagulants is reported presents challenges for quantitative analysis and reproducibility. While some reports provided the specific quantity of leaves and/or stems used, others mention only the combined quantity of plant material and solvent (such as milk or water). Future studies could be directed toward understanding the impact of varying coagulant quantities on the characteristics of WASC.

### 3.3 Yield, Physicochemical and Nutritional Composition of WASC

#### 3.3.1 WASC Yield

**FIGURE 5:**
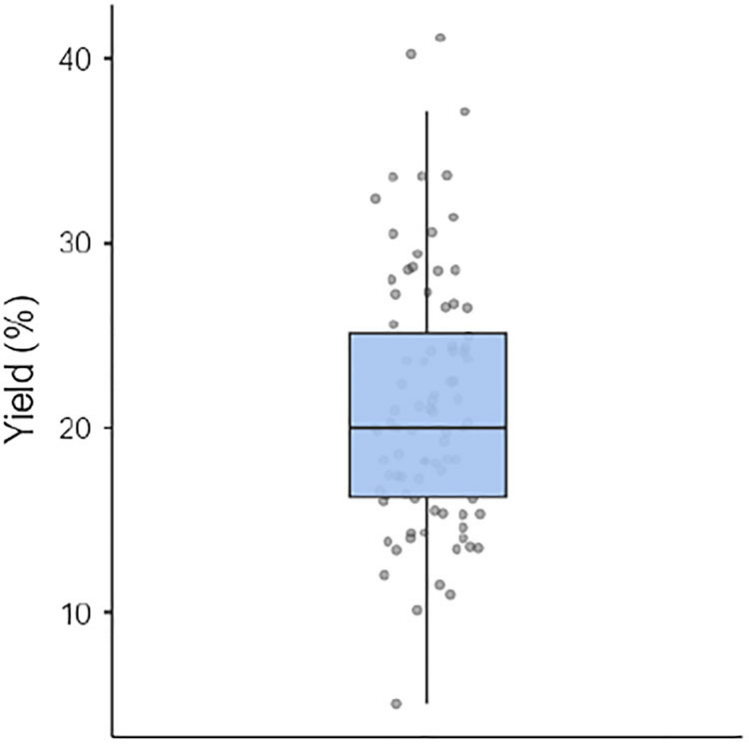
Yield of West African soft cheese according to the literature (*n* = 22).

Cheese yield is a critical factor in the economics and profitability of dairy industries (Mohsin et al. 2024). For WASC, yields reported in the literature range from as low as 5.10% (Ogunlade et al. 2020) to as high as 41.1% (Okorie and Adedokun 2013), with a median of 20% (Figure 5). This remarkable variability of yield points toward a huge potential once the source of it is well understood.

Various factors could influence cheese yields, such as the type and quantity of coagulant used; the milk type, including protein content (particularly when partially replaced with plant‐based alternatives); and the heating temperature during processing (Anema 2021; Mazorra‐Manzano et al. 2018; Mohsin et al. 2024). This review could not definitively establish their specific effects on WASC due to the complexity and heterogeneity of the available data. However, the type of coagulant used and partial replacement of cow's milk seem to have an impact on WASC.

Okorie and Adedokun (2013) reported the highest yield (41.1%), likely due to substituting 50% of cow's milk with Bambara groundnut milk; this formulation produced a significantly higher yield than the control with 100% cow's milk (28%). Okorie and Adedokun (2013) found a linear increase in WASC yield as Bambara groundnut milk incorporation level increased from 5% to 50%, partly attributable to a corresponding increase in protein content from 7.9% (100% cow's milk) and 8.1% (5% Bambara) to 13.6% (50% Bambara). Certain plant‐based milks, such as Tiger nut milk, blended with cow's milk, might significantly enhance yield. For instance, Tiger nut milk inclusion level from 25% to 50% resulted in an increase in WASC yield (Oni et al. 2020). Conversely, coconut milk has been shown to negatively impact WASC yield as the proportion of coconut milk increases (Balogun et al. 2016; Okon and Ojimelukwe 2017; Oni et al. 2020). For example, when *Cal. procera* leaves were used as a coagulant, the lowest yields (13.4% and 13.5%) occurred with 50% (Oni et al. 2020) and 30% (Balogun et al. 2016) replacement of cow's milk, respectively. In contrast, the zero replacement (100% cow's milk) recorded higher yields of 14.3% (Oni et al. 2020) and 26.7% (Balogun et al. 2016) than the formulations with coconut milk. Similarly, Okon and Ojimelukwe (2017) reported that a 30% coconut milk replacement reduced yield to 15.3%, compared to 27.2% with 100% cow's milk.

Soya milk, however, seems to have a lesser impact on cheese yield (Aworh et al. 1987; Chikpah et al. 2016). Aworh et al. (1987) observed that replacing cow's milk with soya milk by up to 20% in WASC processing had no significant adverse effect on the yield (18.6% vs. 17.4%). Similarly, Chikpah et al. (2016) reported comparable yields (21% vs. 19.84%) when cow's milk was replaced with soya milk up to 75%.

Despite these findings, inconsistencies in experimental conditions (studies conducted under varying conditions), varying coagulant concentrations, milk sources, and processing temperatures made comparison across the studies difficult. Thus, more comprehensive and standardized studies are needed to draw definitive conclusions and to provide recommendations for dairy processors.

Regarding coagulant types, the data obtained in this review do not provide conclusive evidence for a significant influence on yield. However, there have been a few reports of considerable coagulant type or concentration effect. In terms of coagulant dosage effect, WASC yield increased from 28.5% to 33.7% when *Cal. procera* extract was increased from 0.5 to 1.5 mL, but the yield declined to 30.5% when the coagulant dose was increased to 2.5 mL (Ogundiwin and Oke 1983). With coagulant type, millet‐steeped water produced the lowest yield (5.1%), while *Cal. procera* leaves had the highest yield (27.3%), followed by *Car. papaya* leaves (24.2%), *Citrus limon* juice (18.3%), and maize‐steeped water (12%) at a coagulant‐to‐milk ratio of 4 g/L in each case (Ogunlade et al. 2020). In Dari and Appah's (2023) study, *Cal. procera* recorded a relatively higher yield (23.6%) than lime (21.8%) and rennet (19.8%). However, yields achieved with *Car. papaya* as a coagulant had a mean of 24.2% (Akinloye and Adewumi 2014; Ogunlade et al. 2020), which is comparable to the yields obtained with *Cal. procera* (22.36%) and *Mo. oleifera* (22.38%) (Adesina et al. 2022; Mahami et al. 2012), regardless of milk type.

**FIGURE 6:**
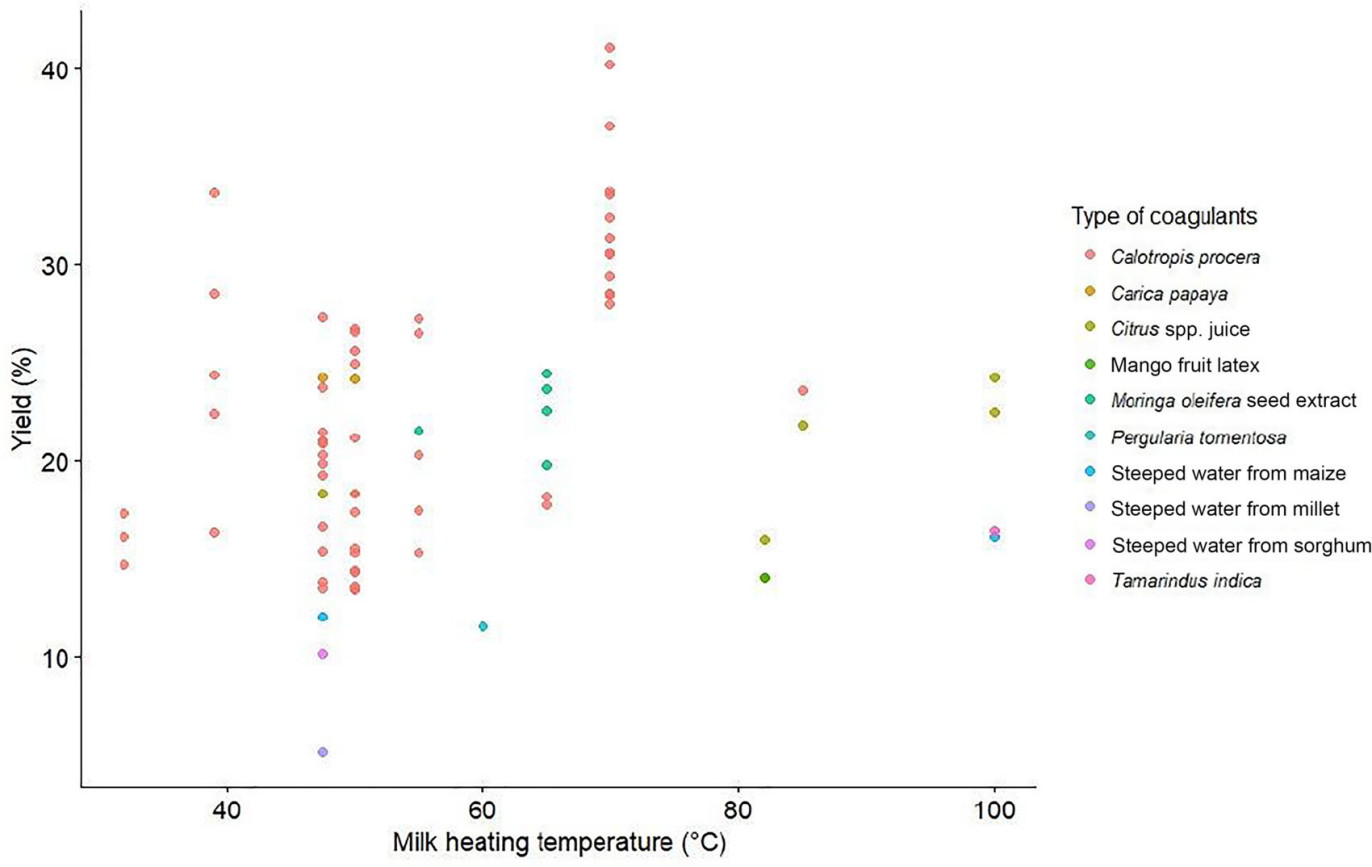
Relationship of West African soft cheese yield per milk heating temperature among the different reported coagulants (*n* = 20).

**FIGURE 7:**
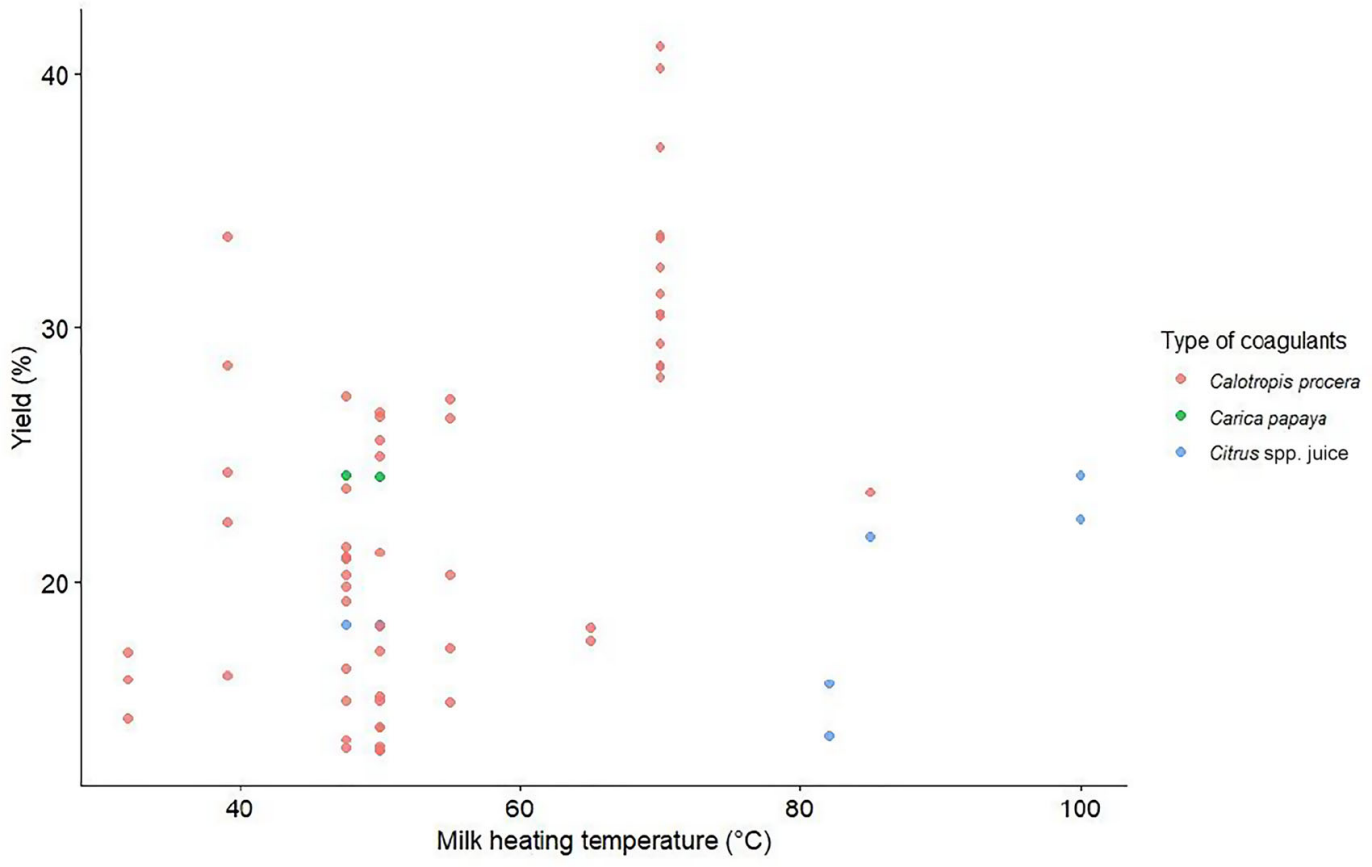
Relationship of West African soft cheese yield per milk heating temperature among the three most reported coagulants (*n* = 18).

*Citrus* spp. yielded between 14% and 20%, with a mean of 19.3% (Akinloye and Adewumi 2014; Ayodeji et al. 2020; Dari and Appah 2023; Olorunnisomo and Adewumi 2016; Ogunlade et al. 2020). By comparison, yields from other coagulants, such as maize‐steeped water (14.1%) (Ogunlade et al. 2020; Ojochogu et al. 2022), *Ma. indica* (14%) (Olorunnisomo and Adewumi 2016), *Pe. tomentosa* (11.5%) (Benyahia et al. 2021), and sorghum‐steeped water (10.1%) (Ogunlade et al. 2020), were generally lower. Such variations are normal, as factors such as enzyme concentration, pH, and the presence of calcium ions influence milk‐clotting activity. These parameters, along with the type and enzymatic properties of plant‐based coagulants, significantly impact their performance (Mazorra‐Manzano et al. 2018; Shah and Mir 2019). Additionally, heating milk before coagulation significantly influences the structure of casein micelles and their interaction with denatured whey proteins, thereby affecting coagulation efficiency (Anema 2021). Figure 6 provides an overview of the relationship between milk heating temperature (°C) and yield (%) across various coagulants, whereas Figure 7 provides an overview of the relationship between milk heating temperature (°C) and yield (%) across the three most reported coagulants (*Cal. procera*, *Car. papaya*, and *Citrus* spp.).

The limited data available for other coagulants prevent comprehensive comparisons, but the findings highlight that the highest yields were observed with *Cal. procera* at approximately 70°C, indicating its effectiveness within this temperature range.

It has been shown that the optimum milk‐clotting activity of *Cal. procera* occurs between 60°C and 70°C (Abebe and Emire 2020; Oseni and Ekperigin 2013). Similarly, papain, derived from the latex of fresh unripe *Car. papaya* fruits, remains highly active over a broad temperature range, with peak activity observed at 60°C and effective milk‐coagulating activity from 20°C to 90°C. However, papain becomes less effective at pH values above 7.0 (Hafid et al. 2020). The leaves of *Car. papaya* have also been reported to contain cysteine protease. The purified enzymes from *Car. papaya* leaves are optimally active from 50°C to 59°C and have a pH ranging from 4.5 to 6.6 (Babalola et al. 2023). Moreover, *Mo. oleifera* flowers exhibit the highest clotting activity when milk is pretreated at 70°C (Pontual et al. 2012). These findings underscore the importance of optimizing milk heating temperature and understanding coagulant‐specific properties to achieve the desired coagulation performance.

**FIGURE 8:**
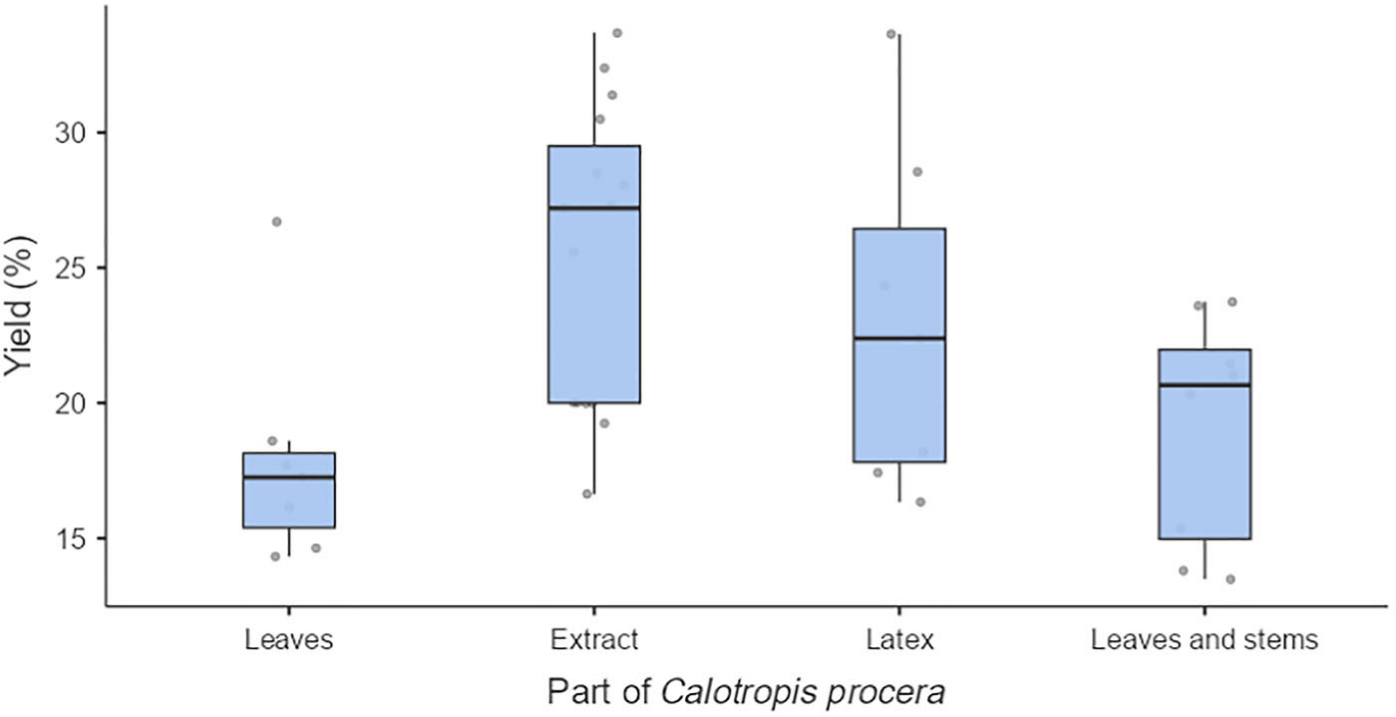
Reported yield of West African soft cheese processed with 100% cow's milk using *Calotropis procera* (*n* = 18).

The variability in milk‐coagulating properties is also influenced by the physiological state of the plant and ecotype variations (Mohsin et al. 2024). As the majority of the data reviewed focus on cow's milk as the primary raw material and *Cal. procera* as the coagulant, we conducted a comparative analysis of the cheese yield based on the specific plant parts utilized (Aworh et al. 1987; Akinloye and Adewumi 2014; Abebe and Emire 2020; Adesina et al. 2022; Balogun et al. 2016; Chikpah et al. 2014; Chikpah et al. 2016; Dari and Appah 2023; Koranteng et al. 2021; Mahami et al. 2012; Ogundiwin and Oke 1983; Oladipo and Jadesimi 2013; Okorie and Adedokun 2013; Ojedapo et al. 2014; Okon and Ojimelukwe 2017; Ogunlade et al. 2020; Oni et al. 2020) as shown in Figure 8.

The comparison of cheese yield obtained using different parts of *Cal. procera* (leaves, extract, latex, and a mixture of leaves and stems) as coagulants reveals differences in performance. WASC yields from leaves ranged from 14.3% to 26.7% (*M* = 17.9%) and exhibited relatively low variability, as reflected by the narrow interquartile range (IQR = 2.76). This consistency suggests that leaves reliably produce modest yields. In contrast, the extract had the highest yields among the four categories, ranging from 16.6% to 33.7% (*M* = 25.4%), but with substantial variability (IQR = 9.5) and several outliers. This variability could be attributed to differences in extraction methods and enzyme concentrations, indicating inconsistent results. Yields from the latex (16.3%–33.6%, *M* = 23%) are comparable to those of the extract and also displayed significant variability (IQR = 8.6). This wide range suggests inconsistency, likely due to differences in the quantity used. The yields from the mixture of leaves and stems (13.5%–23.7.6%, *M* = 19.1%) were quite comparable to those of the extract and the latex. Although the variability (IQR = 6.99) is lower than that of the extract and latex, it is still greater than that of leaves alone. Regardless, these yields are relatively higher than the 8.9%–9.4% reported for cheddar cheese, coagulated using rennet (Soodam et al. 2015), but similar to the 18.8%–20.0% found for Pakistani cottage cheese (Ali et al. 2022).

Several questions arise regarding the factors contributing to the observed variations in the yield of WASC when using *Cal. procera* as a coagulant. It is not emphatic as to whether the quantity of the coagulant is the sole determinant for these variations, or whether other factors, such as the physiological characteristics and ecological variability of the plant, also play a significant role. These uncertainties highlight the need for a deeper understanding of the variables influencing the performance of *Cal. procera* and other plant‐based coagulants. Future research could adopt a multidisciplinary approach to optimize the use of *Cal. procera* and similar plant‐based coagulants. By addressing these gaps in knowledge, it may be possible to enhance their efficiency, consistency, and suitability for large‐scale cheese production.

#### 3.3.2 Physicochemical Properties and Macro‐ and Micronutrients

Due to the lack of standardization in WASC production, its composition can vary significantly. Additionally, factors such as the breed of cattle providing the milk, the microflora present in the milk, the type of coagulant used, and the type of preservative used can lead to variations in the physicochemical and microbiological characteristics of WASC (Sessou et al. 2013; Tougan et al. 2021). Processors attribute the quality of WASC to the quality of the raw milk and critical processing steps, particularly coagulation and cooking (Dossou et al. 2022). Respondents emphasized keeping equipment hygienic, preventing any milk–salt/water contact, and using fresh *Cal. procera* leaves or stems as key measures (Dossou et al. 2022). Moreover, the partial substitution of cow's milk with plant‐based or alternative milk sources leads to mixed changes in its physicochemical characteristics (Okorie and Adedokun 2013). WASC exhibits a broad compositional diversity, providing essential nutrients in varying amounts, which could address specific dietary needs and play a significant role in enhancing nutritional security, particularly in low‐resource settings. The pH of WASC ranged from 4.2 to 6.8 (Adetunji et al. 2008; Akinloye and Adewumi 2014; Abebe and Emire 2020; Aboudoulaye and Kaya 2020; Arthur et al. 2023; Dossou et al. 2024; Hussein et al. 2016; Okon and Ojimelukwe 2017), which can affect its shelf life as the pH plays an important role in controlling microbial growth in food. This pH range is identical to a range of 4.4–6.7 reported for some cottage cheeses (Abdeen et al. 2024; Ali et al. 2022; Nair et al. 2022).

This review provides a comprehensive overview of the moisture, protein, fat, and ash contents retrieved from 28 studies (Adegoke et al. 1992; Adetunji and Salawu 2008; Ayeni et al. 2014; Adeyeye 2017; Abebe and Emire 2020; Aboudoulaye and Kaya 2020; Ayodeji et al. 2020; Adesina et al. 2022; Balogun et al. 2016; Benyahia et al. 2016; Badmos et al. 2018; Bankolé et al. 2019; Chikpah et al. 2016; Hussein et al. 2016; Koranteng et al. 2021; Lawal and Adedeji 2013; Mahami et al. 2012; Ogundiwin and Oke 1983; Okorie and Adedokun 2013; Oladipo and Jadesimi 2013; Ojedapo et al. 2014; Omobolanle et al. 2015; Olorunnisomo and Adewumi 2016; Orhevba and Taiwo 2016; Okon and Ojimelukwe 2017; Oni et al. 2020; Ojochogu et al. 2022; Tossou et al. 2018). These four proximate components are reported because they were dominantly measured. Most of the studies lacked the complete proximate composition, hence settling on the most determined.

**FIGURE 9:**
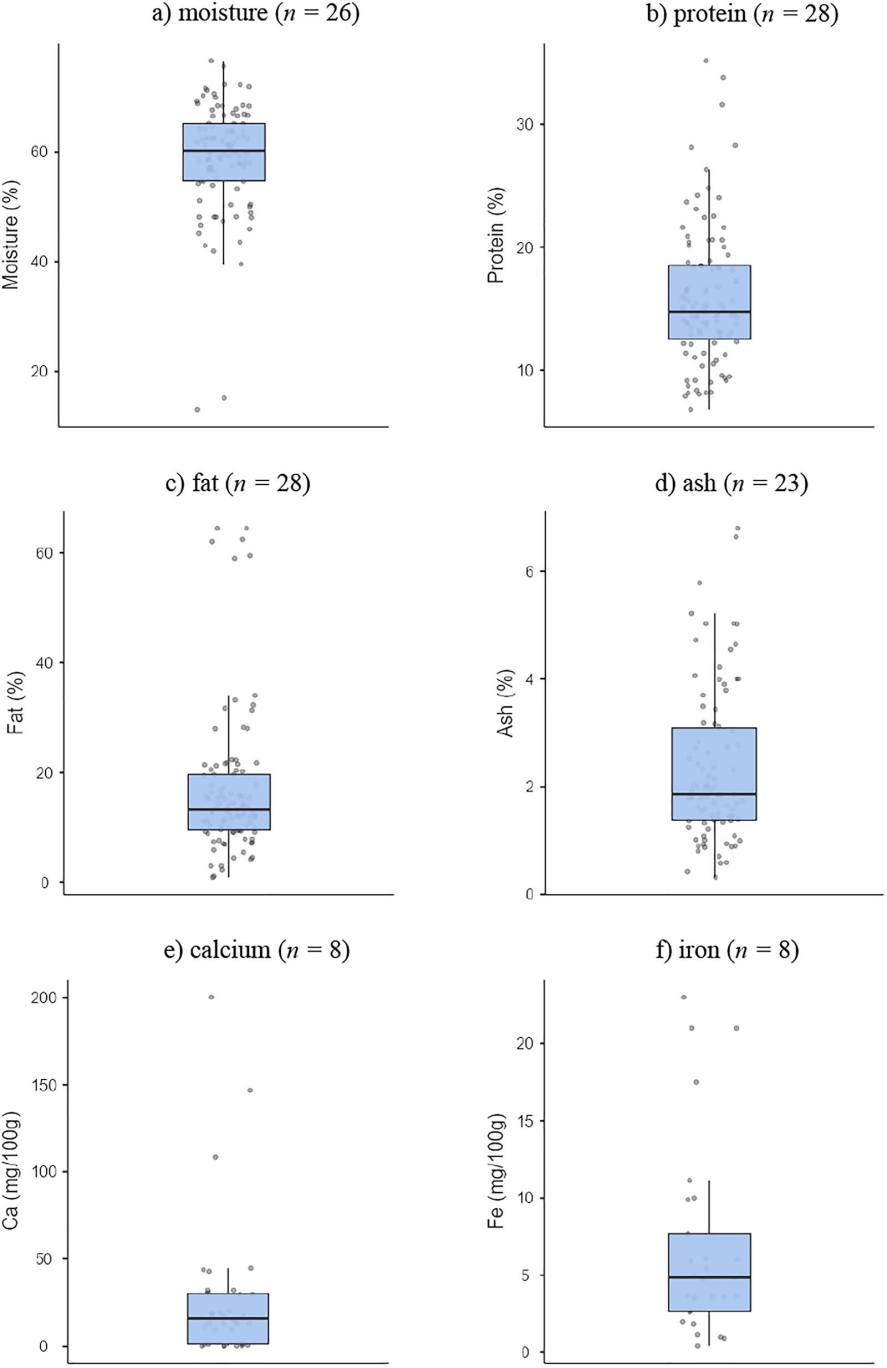
Reported physicochemical, nutritional, and mineral composition of West African soft cheese (% related to fresh weight).

The reported moisture content of WASC ranged from 13.1% to 76.6%, with a median of 60.8%. Two unusually low moisture values (13.1% and 15.1%) (Figure 9a) were reported for cheeses produced using maize‐steeped water and *T. indica* extract as coagulants, respectively (Ojochogu et al. 2022). These values fall below the typical range for soft cheeses, classifying them as hard cheeses (moisture <25%) (McSweeney et al. 2004). These values are even lower than the moisture content of cheeses like cheddar (37%–41%) (Soodam et al. 2015; Renner 1997), blue cheese (43%), *parmesan* (31%), *emmentaler* (36%), and *tilsiter* and *gouda* (46%) (Renner 1997). However, upper limit values are quite close to the moisture content of cheeses from other studies: feta (63%), fresh cheese (75%), and cottage cheese (64%–80%) (Eshetu and Asresie 2019; Renner 1997; Sharma et al. 2018). Incorporating soya milk into cheese formulations was shown to increase moisture content proportionally with the incorporation rate (Chikpah et al. 2016; Hussein et al. 2016).

**FIGURE 1:**
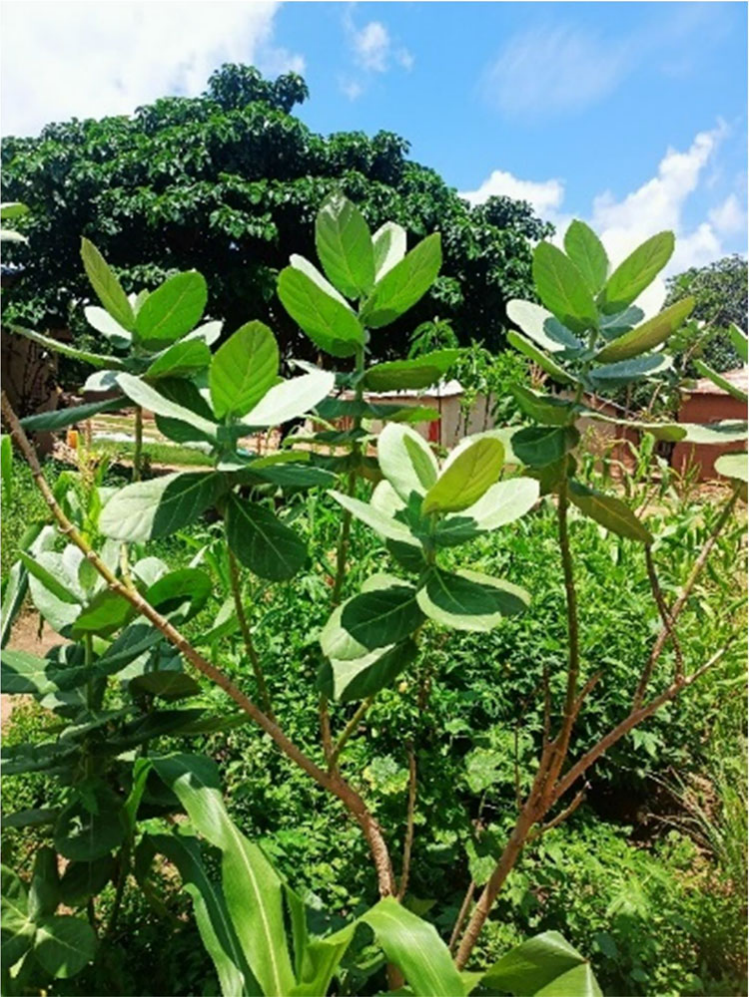
*Calotropis procera* plant. Credit: Mahounakpon, 2023.

**FIGURE 2:**
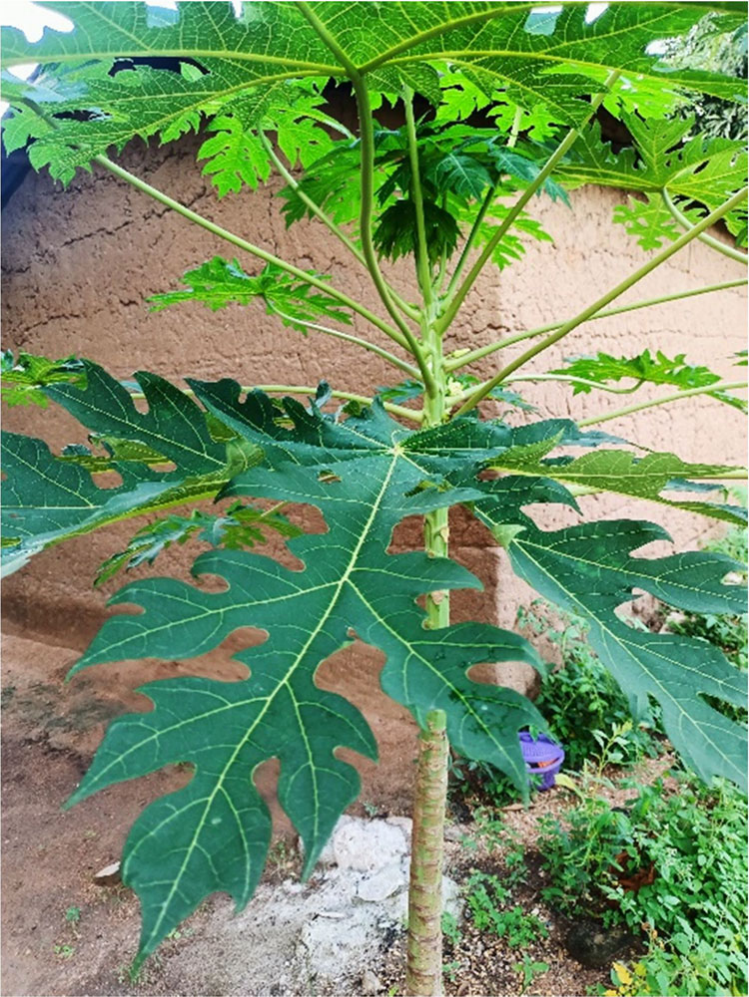
*Carica papaya* plant. Credit: Mahounakpon, 2023.

The protein content varied widely, from 6.8% (Oladipo and Jadesimi 2013) to 35.2% (Adeyeye 2017), with a median of 14.7%, demonstrating the potential of WASC as a high‐protein food (Figure 9b). Incorporating plant‐based milk, such as coconut milk, enhanced protein content, with increases ranging from 9% to 21% at a 40% incorporation rate (Omobolanle et al. 2015) and similar improvements at a lower rate (30%) (Balogun et al. 2016; Okon and Ojimelukwe 2017). Tiger nut milk also increased the protein content from 16.8% to 18.8% at a 25% incorporation rate (Oni et al. 2020). Additionally, Bambara groundnut increased the protein content from 7.9% to 13.6% at a 50% incorporation rate (Okorie and Adedokun 2013). The protein values found in this review are somewhat similar to the 14.5%–17.8% reported for some cottage cheeses elsewhere in Ethiopia and India (Eshetu and Asresie 2019; Sharma et al. 2018) but slightly lower than the 40.8%–41.5% reported for fermented cottage cheese (*Metata*) in Ethiopia (Eshetu and Asresie 2019).

WASC features fat content ranging from 1.02% (Oladipo and Jadesimi 2013) to 64.5% (dry weight basis) (Orhevba and Taiwo 2016), with a median of 13.3% (Figure 9c); the lowest value could be due to the benzoate treatment used in that study (Oladipo and Jadesimi 2013). While plant‐based milk incorporation generally increased protein, the contrast was observed for fat contents. For example, Bambara groundnut and soya milk substitutions significantly reduced the fat content with increasing incorporation rates (Chikpah et al. 2016; Okorie and Adedokun 2013). With more interest being shifted toward consuming less‐fat foods, WASC incorporated with plant‐based milk could meet some consumer needs and dietary preferences. Fat contents of about 1.4% (*Ayib* and *Hazo* cheeses) and 30.1%–31.9% (*Metata*) were reported by Eshetu and Asresie (2019), 29.1% for cream cottage cheese (Abdeen et al. 2024), and 22.4%–24.5% for cheddar cheese (Soodam et al. 2015).

Ash content was reported between 0.32% and 6.8%, with a median of 1.86% (Figure 9d). This range is comparable to the 1.1%–6.9% ash content found in similar cheeses (Eshetu and Asresie 2019; Nair et al. 2022). WASC serves as a vital source of minerals, particularly iron and calcium, which are critical for bone health, oxygen transport, and enzymatic functions (Bechthold et al. 2019).

The iron content of WASC ranged from 0.44 to 23 mg/100 g, with a median of 4.84 mg/100 g (Figure 9f) (Adetunji and Salawu 2008; Ayeni et al. 2014; Abebe and Emire 2020; Chikpah et al. 2016; Lawal and Adedeji 2013; Okorie and Adedokun 2013; Omobolanle et al. 2015; Okon and Ojimelukwe 2017). The highest iron contents obtained were due to the incorporation of coconut milk (Omobolanle et al. 2015; Okon and Ojimelukwe 2017).

Calcium content ranged from 0.3 to 200 mg/100 g, with a median of 15.8 mg/100 g (Figure 9e) (Abebe and Emire 2020; Chikpah et al. 2016; Koranteng et al. 2021; Lawal and Adedeji 2013; Okorie and Adedokun 2013; Omobolanle et al. 2015; Orhevba and Taiwo 2016; Okon and Ojimelukwe 2017). This remarkable variability in composition underscores the need for further research to optimize cheese production methods. Future studies should particularly focus on the effects of partial milk substitution and alternative coagulants on the physicochemical and nutritional properties of WASC to ensure product quality and consistency. Furthermore, this variability should be considered when estimating the nutrient intake from animal‐sourced foods in dietary studies.

### 3.4 Microbial Evaluation and Safety of WASC

The WASC is frequently associated with poor microbial quality, irrespective of its nutritive and sensorial value, due to hygiene‐related issues, primarily inadequate hygiene practices during production, handling, and distribution. These issues encompass the entire value chain, from the production process to the conditions under which the cheese is sold. Commonly sold either unpackaged at ambient temperatures or packaged in plastic bags, the cheese is marketed through stationary retail outlets or via mobile vendors on streets and in marketplaces (Dauda 2017; Dauda et al. 2018; Kunadu et al. 2018). Consumers often perceive indigenous dairy products as unsafe, a perception largely influenced by observable deficiencies in environmental and personal hygiene surrounding the producers (Kunadu et al. 2019).

Of the studies included in this review, 17 focused on the safety of WASC, with 13 involving sample analyses and four experiments (Adegoke et al. 1992; Adetunji et al. 2008; Adetunji and Arigbede 2011; Adetunji and Chen 2011; Adeyeye 2017; Abebe and Emire 2020; Arthur et al. 2023; Belewu and Aina 2000; Dossou et al. 2024; Kunadu et al. 2018; Kunadu et al. 2019; Kortei and Annan 2022; Kortei et al. 2024; Mwini and Darkwa 2016; Modupe et al. 2019; Okon and Ojimelukwe 2017; Owolabi et al. 2023).

While the quality of the raw milk is very important, the coagulation stage (i.e., use of *Cal. procera* extract as a coagulant) was identified as a critical control point in cheese production (Adetunji and Arigbede 2011). The addition of *Cal. procera* introduces contaminants into the cheese, as evidenced by an increase in microbial load (aerobic plate microbes) from 6.5 log_10_ CFU/g in raw milk to 7.4 log_10_ CFU/g after adding the extract (Adegoke et al. 1992). However, subsequent cooking reduced the microbial load to 1.7 log_10_ CFU/g, indicating that adequate thermal treatment can enhance product safety. The dominant microbial flora detected included *Mucor* spp. and *Bacillus* spp. (Adegoke et al. 1992).

Multiple studies documented unsatisfactory hygienic practices and significant microbial contamination in WASC. Belewu and Aina (2000) reported high counts of aerobic plate microbes (9.7 log_10_ CFU/g), *Escherichia coli* (8.5 log_10_ CFU/g), *Enterococcus* spp. (6.9 log_10_ CFU/g), and *Klebsiella* spp. (8.5 log_10_ CFU/g) in WASC samples from vendors in Nigeria. Moreover, the presence of pathogens such as coliforms, *Bacillus* spp., and *Mucor* spp. highlighted inadequate sanitation, postproduction contamination, and unhygienic handling during vending (Belewu and Aina 2000). Those results are rather high if compared with the Ghana Standards 955 (Ghana Standards Authority 2018). Acceptable microbial limits for WASC and other cheeses include a maximum of 3 log_10_ CFU/g for aerobic plate counts and 2 log_10_ CFU/g for *Es. coli* and *Staphylococcus aureus*, with no detectable *Salmonella* spp. or *Listeria* spp. (Arthur et al. 2023).

Adetunji and Arigbede (2011) found that raw milk samples from local factories in Nigeria were already contaminated by *Listeria monocytogenes* (6.3 log_10_ CFU/g) and *Es. coli* (7 log_10_ CFU/g), whose final loads were 8.4 and 9.2 log_10_ CFU/g in the cheese, respectively, after processing. Though *Li. monocytogenes* has some heat resistance, the higher loads could partly be explained by recontamination from contaminated strainers and/or the environment. Similar observations were made by Kunadu et al. (2018) in Ghana, where fresh milk samples with an initial microbial load of 7.5 log_10_ CFU/g led to cheese contamination levels as high as 10.1 log_10_ CFU/g. However, *Es. coli* O15:H7 and *Salmonella enterica*, initially undetermined in raw milk, were found in cheese; this could be a result of unhygienic handling.

Adeyeye (2017) detected the presence of *Campylobacter jejuni* (6.2 log_10_ CFU/g) and *Salmonella* spp. (2.7 log_10_ CFU/g) in WASC sold in Nigerian streets, reflecting poor hygiene in handling and vending. Arthur et al. (2023) recorded high aerobic mesophilic counts in fresh (9.6 log_10_ CFU/g) and fried (6.8 log_10_ CFU/g) WASC samples from Ghanaian markets, alongside coliform counts of 5.4 and 3.3 log_10_ CFU/g, respectively.

Modupe et al. (2019) provided further evidence of WASC's poor microbial quality in Nigerian markets. Cheese samples exhibited aerobic plate counts of 5.6 log_10_ CFU/g and were contaminated with a variety of bacteria and fungi. Pathogens isolated included *Klebsiella edwardsii*, *Streptococcus* spp., *Enterococcus faecalis*, *Lactobacillus acidophilus*, *Bacillus cereus*, *Pseudomonas putida*, *Shigella* spp., *Pseudomonas fluorescens*, *Es. coli*, and *St. aureus* (Adetundji and Adegoke 2007;Modupe et al. 2019). These results underscored the serious deficiencies in hygiene and sanitation during processing and marketing. The results also exposed poor regulatory checks and monitoring by the regulatory bodies.

In contrast to market and/or local factory samples, WASC produced under controlled laboratory conditions exhibited significantly improved safety profiles. For instance, Abebe and Emire (2020) reported an aerobic plate count of 3.3 log_10_ CFU/g with no detectable *Es. coli*. Similarly, Ayodeji et al. (2020) documented an aerobic plate count of 2.9 log_10_ CFU/g, while Okon and Ojimelukwe (2017) reported a lower aerobic count of 1.5 log_10_ CFU/g, with no coliforms detected. Adetunji et al. (2008) reported an aerobic plate count of 2.1 log_10_ CFU/g using lemon juice as a coagulant.

Furthermore, the presence of high concentrations of aflatoxins in WASC, specifically aflatoxin B1 (AFB1) (4.8–5.3 µg/kg) and aflatoxin M1 (AFM1) (6.4–7.5 µg/kg) (Adegoke et al. 1992), was alarming, considering that the Codex Alimentarius maximum allowable level for AFM1 in raw milk is 0.5 µg/kg (FAO and WHO 2009). A study by Dossou et al. (2024) detected AFM1 levels of 0.3 µg/kg in cheese samples from Benin. Additionally, studies by Kortei and Annan (2022) and Kortei et al. (2024), detecting AFM1 levels of 1.8 and 0.03 µg/kg, respectively, indicate variations across regions and processing conditions.

These microbial safety issues are not just peculiar to WASC alone; other cheeses also face this canker. For instance, Rebić et al. (2023) reported that about 45% of forty types of cheeses sampled in Bosnia and Herzegovina failed to meet microbial safety standards. Similarly, about 61%–98% of cottage cheese samples in Ethiopia were found to be contaminated with unsafe levels of different kinds of pathogens (Keba et al. 2024). Al‐Groom (2017) found 10%–14%, 8%–12%, and 8%–10% of cheddar cheese samples collected from markets and restaurants in Amman, Jordan, were contaminated with coliform, *St. aureus*, and *Salmonella*, respectively.

Improving the safety of WASC requires a multifaceted approach. This includes sourcing milk from healthy animals, ensuring hygienic milking practices, thoroughly washing *Cal. procera* leaves and/or stems before juice extraction, and closely monitoring processing temperatures. Pasteurization of the milk before WASC processing can also be an important step to reducing microbial loads, most especially pathogens, in the final product (Johnson et al. 1990; Molina 2023). Enhanced education and training of producers and vendors in proper hygiene practices could also play a critical role in mitigating postprocessing contamination risks (Achaglinkame et al. 2023). Effective programs should cover (i) personal hygiene, (ii) utensil and surface sanitation, (iii) water quality, (iv) time–temperature control, and (v) hygienic packaging and display. The provision of low‐cost enabling supplies (such as soap, sanitizers, and food‐grade liners), peer mentoring through producer associations, and regular inspections will further strengthen these programs.

### 3.5 Packaging, Conservation, and Storage of WASC

WASC has, under ambient conditions, a short shelf life of approximately 2–3 days without additional preservation methods (Adegoke et al. 1992). Given the absence of household refrigeration and unreliable electricity supply in rural West African regions, various efforts have been made to prolong the WASC's shelf life. While most of the preservative methods to enhance the shelf life are traditional (boiling, drying, smoking/roasting, etc.), studies have looked at chemical preservation (sorbic acid and sodium benzoate) (Aworh and Egounlety 1985; Ashaye et al. 2006; Alalade and Adeneye 2007; Adetunji et al. 2008; Alalade and Adeneye 2010; Adetunji and Chen 2011; Akogou et al. 2018; Arthur et al. 2023; Belewu et al. 2005; Badmos et al. 2017; Badmos et al. 2018; Dossou et al. 2022; Joseph and Akinyosoye 1997; Malomo et al. 2020; Mazou et al. 2012; Oladipo and Jadesimi 2013; Ogboju et al. 2018; Sanni et al. 1999; Saliu et al. 2014).

A survey carried out by Dossou et al. (2022) among 390 actors (84 dairy farmers, 165 producers, 53 traders, and 88 consumers) from two municipalities (Dassa and Nikki) in Benin revealed six traditional methods of WASC preservation. These methods include (i) boiling the cheese in water, salt solution, or shea leaves–water mixture, either directly or packaged in plastic bags; (ii) immersing the cheese in whey or water for short‐term preservation; (iii) drying the cheese in the open sun; (iv) coloring the cheese with natural dyes such as sorghum cobs/panicles by soaking or boiling; (v) smoking; and (vi) frying (Dossou et al. 2022). Immersing WASC in the whey has been shown to preserve it for up to 3 days at room temperature (28°C) with good hygienic practices (Alalade and Adeneye 2010). Additionally, sun‐drying, heating in salt solutions, cold storage, and roasting have been reported to reduce microbial activity, thus improving the shelf life of WASC (Alalade and Adeneye 2007; Dah et al. 2018; Mazou et al. 2012). However, this is only an inference, as no data indicating how long the aforementioned processes extend the shelf life of the cheese under discussion currently exist. Boiling cheese in salted water was more effective than either boiling without salt or salting alone in preserving WASC (Saliu et al. 2014; Sanni et al. 1999). Moreover, Arthur et al. (2023) reported that deep frying (110°C for 2 min) combined with aseptic packaging reduced total aerobic counts from 8.4 to 2.7 log_10_ CFU/g and eliminated contaminants like *Enterobacteriaceae*, coliforms (as *Es. coli*), yeasts, and molds. It is, however, unclear whether the individual factors had any effect on the microbial counts and the proximate composition of WASC.

The use of dyes is common in Benin and other African regions. For example, dye from sorghum extract is traditionally valued in the West African region not only for its role in coloring soft cheese but also for its wider applications, such as coloring porridges and foods (Kayodé et al. 2011). The dyeing process is publicly perceived as a preservation method and a strategy to increase market appeal (Akogou et al. 2018). However, studies reveal that sorghum biocolorant does not inhibit the growth of spoilage fungi (*Penicillium chrysogenum*, *Cladosporium macrocarpum*) or pathogens like *Es. coli* O157:H7 and *Li. monocytogenes* in cheese or broth cultures (Akogou et al. 2018; Joseph and Akinyosoye 1997). Interestingly, while sorghum dye does not affect the physicochemical properties of WASC, its application may obscure visible signs of spoilage due to its intense red coloring, potentially misleading producers’ and consumers’ perceptions (Akogou et al. 2018).

The use of lemon juice as a coagulant when processing the WASC also appears as another way to improve its shelf life. Adetunji et al. (2008) and Adetunji (2011) demonstrated that lemon juice significantly reduced populations of *Enterobacteriaceae*. In experiments involving milk initially inoculated with *Li. monocytogenes* at 1 and 2 log_10_ CFU/mL, the population decreased to an undetectable level (<1 log_10_ CFU/g) with the addition of lemon juice. Additionally, *Enterobacteriaceae* counts decreased from 1.8 and 3.6 log_10_ CFU/g in the milk to undetectable levels (<1 log_10_ CFU/g) at the curdling stage. This inhibitory effect persisted during a 5‐day storage period at 15°C and 28°C (Adetunji et al. 2008). These findings highlight the dual functionality of lemon juice as both a coagulant and a natural antimicrobial agent, offering a simple and cost‐effective strategy for improving the safety and shelf life of WASC. However, further studies are needed to evaluate the effectiveness of lemon juice across varied formulations and storage conditions to establish robust evidence.

Ginger, garlic, and natural extracts have shown considerable prospects in preserving WASC. Belewu et al. (2005) demonstrated the potential of preservation by dipping or boiling WASC with ginger and/or garlic extract. Additionally, Malomo et al. (2020) investigated the preservative effect of ginger and sorbic acid and recommended a combination of 2.5% ginger with 0.05% sorbic acid, which extended shelf life without severely compromising sensory properties such as odor and taste. Garlic and ginger extracts delayed the spoilage of WASC to 13 and 15 days, respectively, under refrigeration conditions (6°C). Badmos et al. (2017) highlighted that WASC supplemented with ether extracts of the spice *Aframomum melegueta* powdered seeds at 3% inhibited microbial growth and increased fat, ash, and protein contents. However, the use of the extracts adversely affected the sensory properties.

Chemical preservatives such as sorbic acid and sodium benzoate are widely recognized for their effectiveness. Aworh and Egounlety (1985) showed that sorbic acid, at 0.1% concentration, inhibited the growth of mesophiles in WASC stored at 7°C–9°C. Oladipo and Jadesimi (2013) found that 0.5% sodium benzoate extended shelf life from 4 to 11 days under refrigeration conditions (6°C).

Introducing vacuum packaging has emerged as another effective technique to extend the shelf life of WASC. Adetunji and Chen (2011) reported that vacuum‐packaged cheese samples exhibited no detectable yeast or mold growth after 21 days at 28°C, compared to 8.4 log_10_ CFU/g in nonpackaged samples stored for 5 days. However, its implementation remains challenging for the small‐scale processors, who are mostly in rural communities.

Efforts to extend the shelf life of WASC continue to evolve, blending traditional practices with modern innovations. Leveraging challenges such as low patronage of dairy farming, inadequate pro‐dairy policies, and an unorganized local sector could help improve West African production, safety, and marketing of WASC (Achaglinkame et al. 2023). Future research should focus on integrating these methods into scalable, cost‐effective solutions that address the unique challenges faced by rural producers, ensuring both safety and sustainability in West African cheese production.

### 3.6 Sensory Properties and Consumer Acceptance of WASC

The sensory evaluation of WASC typically involves a comprehensive assessment of various parameters, including appearance, texture, flavor, color, odor, aroma, and taste. These evaluations are typically conducted using hedonic scales, either 5‐ or 9‐point (Abebe and Emire 2020; Dari and Appah 2023; Oladipo and Jadesimi 2013; Saliu et al. 2014). Existing sensory studies have primarily focused on evaluating how preservative techniques or ingredient substitutions affect consumer acceptability. For instance, while assessing the preservative effects of aqueous and ether extracts of *Aframomum melegueta* on WASC, Badmos et al. (2017) observed that cheeses treated with the extract were the least preferred by panelists due to their unfavorable sensory attributes (color, taste, aroma). Similarly, partial substitution of cow's milk has shown significant effects on sensory parameters. Chikpah et al. (2016) found that increasing the inclusion rates of soya milk in WASC production significantly reduced the color, flavor, and taste liking of the cheese. Blends of cow milk and up to 10% coconut milk produced an overall acceptable WASC (Balogun et al. 2016). Furthermore, Okon and Ojimelukwe (2017) demonstrated that incorporating up to 30% coconut milk yielded products with favorable sensory properties. Soya milk supplementation up to 20% (Aworh et al. 1987) or 25% (Hussein et al. 2016) showed no negative effects on consumer acceptance, while a 25% Tiger nut milk blend was identified as suitable based on consumer acceptability ratings (Oni et al. 2020). Dari and Appah (2023) concluded that *Citrus* spp. and *Cal. procera* provided similar aroma, taste, and mouthfeel using a 5‐point hedonic scale.

More advanced sensory studies have employed quantitative descriptive analysis. In Ghana, Arthur et al. (2023) identified milky, sour, cheesy, salty, and fried egg tastes as potentially desirable attributes of WASC, whereas bitter and bland tastes were deemed undesirable. The list of attributes used with their description is shown in Table 3. A consumer survey conducted in Benin by Dossou et al. (2022) revealed that whiteness, softness, smoothness, and firmness emerged as the most favored attributes. Direct evidence linking specific plant coagulants or raw milk quality to WASC sensory results is scarce; most studies report overall acceptability without isolating these factors. Therefore, further sensory and consumer research is needed to sort out the effects of ingredients and processing. Moreover, research should also focus on characterizing the diversity of preferences among demographic, cultural, and regional groups.

**TABLE 3:** Desirable and undesirable attributes of fresh and fried West African soft cheese developed by a 12‐member panel for quantitative descriptive analysis (Arthur et al. 2023).

Attribute	Desirable	Definition	Undesirable	Definition
Taste	Sour	Taste sensation associated with fermented milk	Bland	Tasteless sensation in a food product
	Cheesy	Taste sensation associated with cheese	Bitter taste	Taste sensation of quinine or caffeine
	Milky	Taste associated with fresh milk		
	Fried egg	Taste associated with omelet		
	Salty	Taste produced by a solution of sodium chloride		
Texture	Soft	Degree of stickiness in the mouth		
	Smooth	The spreadability of the cheese		
	Spongy	The texture characteristics of a bouncy cheese		
	Crumbly	The extent to which the cheese breaks in the mouth		
Aroma	Milky	Aromatics of milk from dairy origin	Spoilt milk	Aromatics of spoilt milk
	Yoghurt	Aromatics of plain yoghurt	Fermented cassava dough	Aromatics of fermented cassava dough
	Cheesy	The aromatic sensation of cheese		
	Beefy	Smell associated with cow meat		
	Fried sweet potato	The smell associated with ripe plantain		
	Doughnut	The smell associated with fried doughnuts		
Color	Whitish	Lighter color		
	Brownish	Dark color as a result of frying		

## 4 Conclusion

The review showed that WASC across West Africa is predominantly produced from cow's milk using *Cal. procera* as the coagulant. The processing steps of the WASC were generally similar across the subregion, with the main variations in the coagulant concentrations used and heating parameters. The existing literature has mainly focused on safety, followed by studies on alternative coagulants and partial substitution of cow's milk. Most of the safety studies reported microbial contamination emanating from unhygienic practices along the value chain. Strengthening good hygiene practices at all stages, complemented by milk pasteurization, should reduce microbial loads, safeguard consumers, and limit postprocessing losses, thereby supporting higher intake, more stable incomes for processors and vendors, and improved food and nutrition security. A range of alternative coagulants has been explored, including *Car. papaya* leaves, *Citrus* spp. fruit juice, *Mo. oleifera* seed extract, *T. indica* fruit extract, unripe *Ma. indica* fruit juice, steeped water from cereals, as well as calcium chloride. Among these, only *Car. papaya* and *Mo. oleifera* were comparable to *Cal. procera* in yield; the rest resulted in significantly lower yields. The findings also revealed that the use of *Citrus* spp. fruit juice as a coagulant extended the shelf life of the cheese. However, comparative records of their effects on the nutritional and sensory qualities of WASC were hardly found. Therefore, further investigations are needed to assess the full impact of those alternatives on yield, microbiological safety, composition, and sensory quality, as well as their scalability and economic feasibility in both artisanal and commercial settings. Regarding the partial cow's milk replacement, the findings showed that increased incorporation levels of Tiger nut milk and Bambara groundnut milk resulted in corresponding increases in the cheese's yield, whereas soya milk and coconut milk decreased it. Partial incorporation of plant‐based milks generally improved the protein content of the cheese but decreased its fat content. Across the evidence base, conclusions about how coagulant type and dose, heating temperature and time, and coagulation/cooking durations influence yield and quality are limited by inconsistent reporting. Future studies should document key processing parameters such as heating temperatures and times, coagulant–milk ratios, coagulation times, and cooking/whey expulsion durations to enable reproducibility and facilitate meta‐analysis. Addressing these reporting gaps, while prioritizing hygiene controls and evidence‐based choices of coagulants and formulations, will offer a practical pathway to safer products and better quality WASC, ultimately reinforcing food and nutrition security without compromising cultural identity.

## Author Contributions

**Mahounakpon Wilfried Tossou**: investigation, writing – original draft, formal analysis. **Matthew Atongbiik Achaglinkame**: investigation, writing – original draft, formal analysis. **Linda Dari**: writing – review and editing, supervision, project administration. **Carole Nadia Adjouavi Sossa‐Vihotogbé**: writing – review and editing, supervision, project administration. **Zsanett Bodor**: formal analysis, writing – review and editing, conceptualization. **Daniel Mörlein**: conceptualization, funding acquisition, supervision, writing – review and editing.

## Conflicts of Interest

The authors declare no conflicts of interest.

## Data Availability

The database used for this review will be made available upon reasonable request.

## Appendix 1 Search queries

### 1.1

Scopus: (TITLE‐ABS‐KEY (west African soft cheese) OR TITLE‐ABS‐KEY (wagashi gassire) OR TITLE‐ABS‐KEY (wangashi) OR TITLE‐ABS‐KEY (warankashi) OR TITLE‐ABS‐KEY (wara)) AND (LIMIT‐TO (LANGUAGE, “English”)) AND (LIMIT‐TO (DOCTYPE, “ar”) OR LIMIT‐TO (DOCTYPE, “cp”))

Web of Science: west African soft cheese (Topic) or wagashi gassire (Topic) or wangashi (Topic) or warankashi (Topic) or wara (Topic) and Article or Other (Document Types) and English (Languages)

PubMed: ((((west African soft cheese) OR (wagashi gassire)) OR (wangashi)) OR (warankashi)) OR (wara) Filters: English
